# Andreev spectrum and supercurrents in nanowire-based SNS junctions containing Majorana bound states

**DOI:** 10.3762/bjnano.9.127

**Published:** 2018-05-03

**Authors:** Jorge Cayao, Annica M Black-Schaffer, Elsa Prada, Ramón Aguado

**Affiliations:** 1 Department of Physics and Astronomy, Uppsala University, Box 516, S-751 20 Uppsala, Sweden; 2 Departamento de Física de la Materia Condensada, Condensed Matter Physics Center (IFIMAC) & Instituto Nicolás Cabrera, Universidad Autónoma de Madrid, E-28049 Madrid, Spain; 3 Instituto de Ciencia de Materiales de Madrid (ICMM-CSIC), Cantoblanco, 28049 Madrid, Spain

**Keywords:** hybrid superconductor–semiconductor nanowire–superconductor junctions, Josephson effect, Majorana bound states, nanowires, spin–orbit coupling, Zeeman interaction

## Abstract

Hybrid superconductor–semiconductor nanowires with Rashba spin–orbit coupling are arguably becoming the leading platform for the search of Majorana bound states (MBSs) in engineered topological superconductors. We perform a systematic numerical study of the low-energy Andreev spectrum and supercurrents in short and long superconductor–normal–superconductor junctions made of nanowires with strong Rashba spin–orbit coupling, where an external Zeeman field is applied perpendicular to the spin–orbit axis. In particular, we investigate the detailed evolution of the Andreev bound states from the trivial into the topological phase and their relation with the emergence of MBSs. Due to the finite length, the system hosts four MBSs, two at the inner part of the junction and two at the outer one. They hybridize and give rise to a finite energy splitting at a superconducting phase difference of π, a well-visible effect that can be traced back to the evolution of the energy spectrum with the Zeeman field: from the trivial phase with Andreev bound states into the topological phase with MBSs. Similarly, we carry out a detailed study of supercurrents for short and long junctions from the trivial to the topological phases. The supercurrent, calculated from the Andreev spectrum, is 2π-periodic in the trivial and topological phases. In the latter it exhibits a clear sawtooth profile at a phase difference of π when the energy splitting is negligible, signalling a strong dependence of current–phase curves on the length of the superconducting regions. Effects of temperature, scalar disorder and reduction of normal transmission on supercurrents are also discussed. Further, we identify the individual contribution of MBSs. In short junctions the MBSs determine the current–phase curves, while in long junctions the spectrum above the gap (quasi-continuum) introduces an important contribution.

## Introduction

A semiconducting nanowire with strong Rashba spin–orbit coupling (SOC) with proximity-induced *s*-wave superconducting correlations can be tuned into a topological superconductor by means of an external Zeeman field [1–3]. This topological phase is characterized by the emergence of zero-energy quasiparticles with Majorana character localized at the nanowire ends. These Majorana bound states (MBSs) are attracting a great deal of attention owing to their potential for topological, fault-tolerant quantum computation [4–6]. Tunneling into such zero-energy MBSs results in a zero-bias peak of high 2*e*^2^/*h* in the tunnelling conductance in normal–superconductor (NS) junctions due to perfect Andreev reflection into a particle–hole symmetric state [7]. Early tunnelling experiments in NS junctions [8–12] reported zero-bias peak values much smaller than the predicted 2*e*^2^/*h*. This deviation from the ideal prediction, together with alternative explanations of the zero-bias peak, resulted in controversy regarding the interpretation. Recent experiments have reported significant fabrication improvements and high-quality semiconductor–superconductor interfaces [13–16] with an overall improvement on tunnelling data that strongly supports the observation of MBS [17–21].

Given this experimental state-of-the-art [22], new geometries and signatures beyond zero-bias peaks in NS junctions will likely be explored in the near future. Among them, nanowire-based superconductor–normal–superconductor (SNS) junctions are very promising since they are expected to host an exotic fractional 4π-periodic Josephson effect [4,23–24], signalling the presence of MBSs in the junction. While this prediction has spurred a great deal of theoretical activity [25–32], experiments are still scarce [33], arguably due to the lack of good junctions until recently. The situation is now different and, since achieving high-quality interfaces is no longer an issue, Andreev-level spectroscopy and phase-biased supercurrents should provide additional signatures for the unambiguous detection of MBSs in nanowire SNS junctions. Similarly, multiple Andreev reflection transport in voltage-biased SNS junctions [34–35] is another promising tool to provide further evidence of MBSs [36].

Motivated by this, we here present a detailed numerical investigation of the formation of Andreev bound states (ABSs) and their evolution into MBSs in nanowire-based short and long SNS junctions biased by a superconducting phase difference 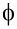. Armed with this information, we also perform a systematic study of the phase-dependent supercurrents in the short- and long-junction limits. Due to finite length, the junction always hosts four MBSs in the topological regime. Apart from the MBSs located at the junction (inner MBSs), two extra MBSs are located at the nanowire ends (outer MBSs). Despite the early predictions [4,23–24] of a 4π-periodic Josephson effect in superconducting junctions containing MBSs, in general we demonstrate that the unavoidable overlap of these MBSs renders the equilibrium Josephson effect 2π-periodic [26–27] in short and long junctions, since they hybridize either through the normal region or through the superconducting regions giving rise to a finite energy splitting at phase difference 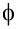 = π. As an example, our calculations show that, for typical InSb parameters, one needs to consider junctions with long superconducting segments of the order of *L*_S_ ≥ 4μm, where *L*_S_ is the length of the S regions, in order to have negligible energy splittings.

In particular, we show that in short junctions with 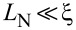, where 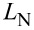 is the normal region length and ξ is the superconducting coherence length, the four MBSs (inner and outer) are the only levels within the induced gap. On the contrary, the four MBSs coexist with additional levels in long junctions with 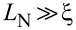, which affect their phase dependence. Despite this difference, we demonstrate that the supercurrents in both limits exhibits a clear sawtooth profile when the energy splitting near 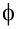 = π is small, therefore signalling the presence of weakly overlapping MBSs. We find that while this sawtooth profile is robust against variations in the normal transmission and scalar disorder, it smooths out when temperature effects are included, making it a fragile, yet useful, signature of MBSs.

We identify that in short junctions the current–phase curves are mainly determined by the levels within the gap and by the four MBSs, with only very little quasi-continuum contribution. In long junctions, however, all the levels within the gap, the MBSs and the additional levels due to longer normal region together with the quasi-continuum determine the current–phase curves. In this situation, the additional levels that arise within the gap disperse almost linearly with 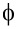 and therefore affect the features of the supercurrents carried by MBSs only.

Another important feature we find is that the current–phase curves do not depend on *L*_S_ in the trivial phase (for both short and long junctions), while they strongly depend on *L*_S_ in the topological phase. Our results demonstrate that this effect is purely connected to the splitting of MBSs at 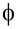 = π, indicating another unique feature connected with the presence of MBSs in the junction. The maximum of such current–phase curves in the topological phase increases as the splitting is reduced, saturating when the splitting is completely suppressed. This and the sawtooth profile in current–phase curves are the main findings of this work. Results presented here therefore strongly complement our previous study on critical currents [37] and should provide useful insight for future experiments looking for Majorana-based signatures in nanowire-based SNS junctions.

The paper is organized as follows. In section “Nanowire model” we describe the model for semiconducting nanowires with SOC, where we show that only the right combination of Rashba SOC, a Zeeman field perpendicular to the spin–orbit axis and *s*-wave superconductivity leads to the emergence of MBSs. Similar results have been presented elsewhere but we include them here for the sake of readability of the next sections. In section “Results and Discussion” we discuss how nanowire-based SNS junctions can be readily modeled using the tools of section “Nanowire model”. Then, we describe the low-energy Andreev spectrum and its evolution from the trivial into the topological phase with the emergence of MBSs. In the same section, we report results on the supercurrent, which exhibits a sawtooth profile at 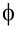 = π as a signature of the emergence of MBSs. In section “Conclusion” we present our conclusions. For the sake of completeness, we also show wavefunction localization and exponential decay as well as homogeneous charge oscillations of the MBSs in wires and SNS junctions in Supporting Information File 1.

## Nanowire model

The aim of this part is to properly describe the emergence of MBSs in semiconducting nanowires with SOC. We consider a single-channel nanowire in one-dimension with SOC and Zeeman interactions, the model Hamiltonian of which is given by [38–43]

[1]
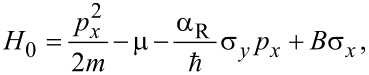


where 
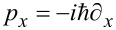
 is the momentum operator, μ the chemical potential that determines the filling of the nanowire, α_R_ represents the strength of Rashba spin–orbit coupling, 
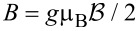
 is the Zeeman energy as a result of the applied magnetic field 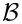 in the *x*-direction along the wire, *g* is the *g*-factor of teh wire and μ_B_ the Bohr magneton. Parameters for InSb nanowires include [8]: the effective mass of the electron, *m* = 0.015*m_e_*, with *m_e_* being the mass of the electron, and the spin–orbit strength α_R_ = 20 meV·nm.

**Figure 1:**
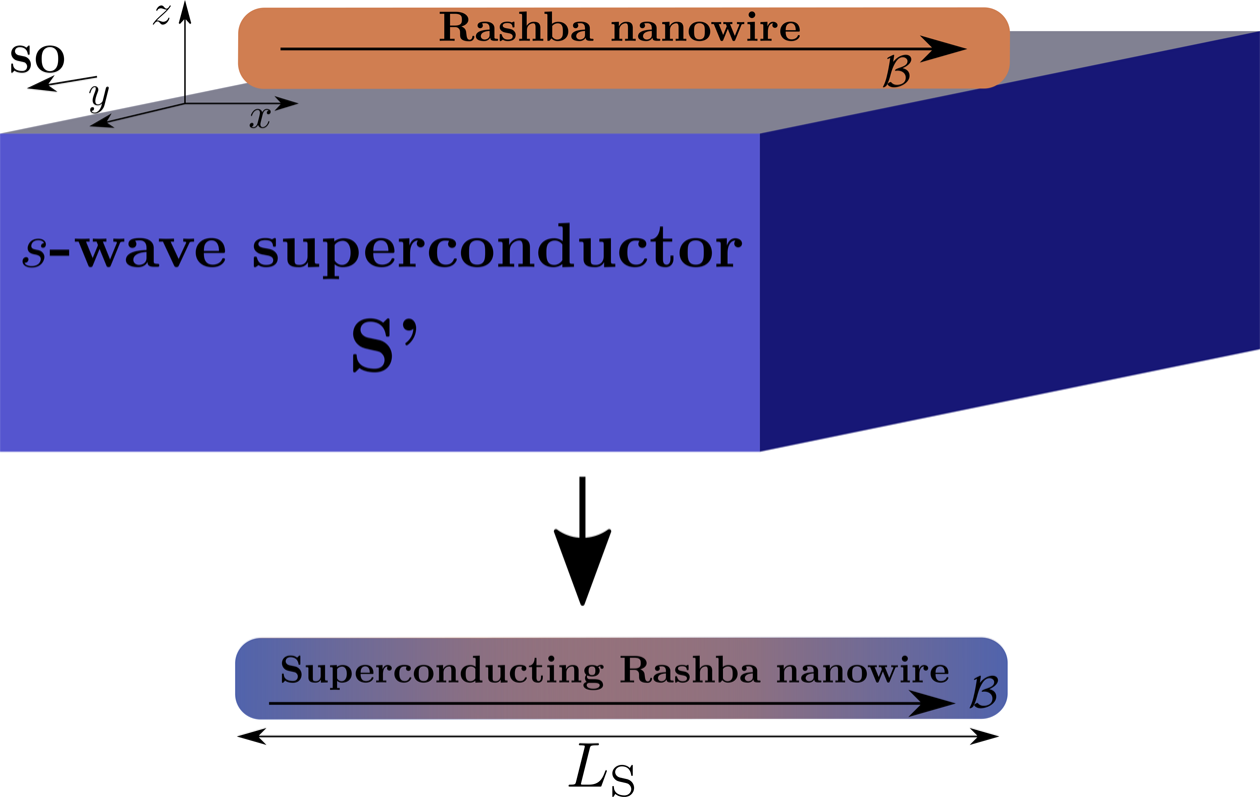
A semiconducting nanowire with Rashba SOC is placed on a *s*-wave superconductor (S’) with pairing potential Δ_S′_ and it is subjected to an external magnetic field 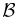 (denoted by the black arrow). Superconducting correlations are induced into the nanowire via proximity effect, thus becoming superconducting with the induced pairing potential Δ_S_ *<* Δ_S′_.

We consider a semiconducting nanowire placed in contact with an *s*-wave superconductor with pairing potential Δ_S′_ (which is in general complex) as schematically shown in Figure 1. Electrons in such a nanowire experience an effective superconducting pairing potential as a result of the so-called proximity effect [44–45]. In order to have a good proximity effect, a highly transmissive interface between the nanowire and the superconductor is required, so that electrons can tunnel between these two systems [13–16]. This results in a superconducting nanowire, with a well-defined induced hard gap (namely, without residual quasiparticle density of states inside the induced superconducting gap). The model describing such a proximitized nanowire can be written in the basis (
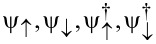
) as

[2]
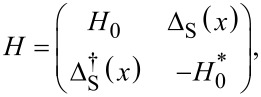


where Δ_S_ *<* Δ_S′_. Since the superconducting correlations are of *s*-wave type, the pairing potential is given by

[3]
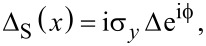


where 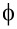 is the superconducting phase. We set 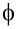 = 0 when discussing superconducting nanowires, while the SNS geometry of course allows a finite phase difference 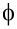 ≠ 0 across the junction.

It was shown [1–2,46] that the nanowire with Rashba SOC and in proximity to an *s*-wave superconductor, described by Equation 2, contains a topological phase characterized by the emergence of MBSs localized at the ends of the wire. This can be understood as follows: The interplay of all these ingredients generates two intraband *p*-wave pairing order parameters


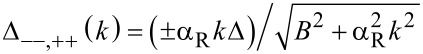


and one interband *s*-wave


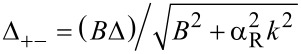


where + and − denote the Rashba bands of *H*_0_. The gaps associated with the ± Bogoliubov–de Gennes (BdG) spectrum are different and correspond to the inner and outer part of the spectrum, denoted by Δ*_1,2_* at low and high momentum, respectively. These gaps depend in a different way on the Zeeman field. Indeed, as the Zeeman field *B* increases, the gap Δ_1_, referred to as the inner gap, is reduced while Δ_2_, referred to as the outer gap, is slightly reduced although for strong SOC it remains roughly constant. The inner gap Δ_1_ closes at *B* = *B*_c_ and reopens for *B > B*_c_ giving rise to the topological phase, while the outer gap remains finite. The topological phase is effectively reached due to the generation of an effective *p*-wave superconductor, which is the result of projecting the system Hamiltonian onto the lower band (−) keeping only the intraband *p*-wave pairing Δ_−−_ [1–2]. Deep in the topological phase *B > B*_c_, the lowest gap is Δ_2_.

In order to elucidate and visualize the topological transition, we first analyze the low-energy spectrum of the superconducting nanowire. This spectrum can be numerically obtained by discretising the Hamiltonian given by Equation 1 into a tight-binding lattice:

[4]
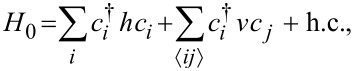


where the symbol 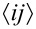 means that *v* couples the nearest-neighbor sites *i*, *j*; *h* = (2*t* − μ)σ_0_ + *B*σ*_x_* and *v* = −*t*σ_0_ + i*t*_SO_σ*_y_* are matrices in spin space, 
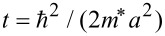
 is the hopping parameter and *t*_SOC_ = α_R_/(2*a*) is the SOC hopping. The dimension of *H*_0_ is set by the number of sites of the wire. Then, it is written in Nambu space as given by Equation 2. Such a Hamiltonian is then diagonalized numerically with its dimensions given by the number of sites *N*_S_ of the wire. Since this description accounts for wires of finite length, it is appropriate for investigating the overlap of MBSs. The length of the superconducting wire is *L*_S_ = *N*_S_*a*, where *N*_S_ is the number of sites and *a* is the lattice spacing. As mentioned before, the superconducting phase in the order parameter is assumed to be zero as it is only relevant when investigating Andreev bound states in SNS junctions.

**Figure 2:**
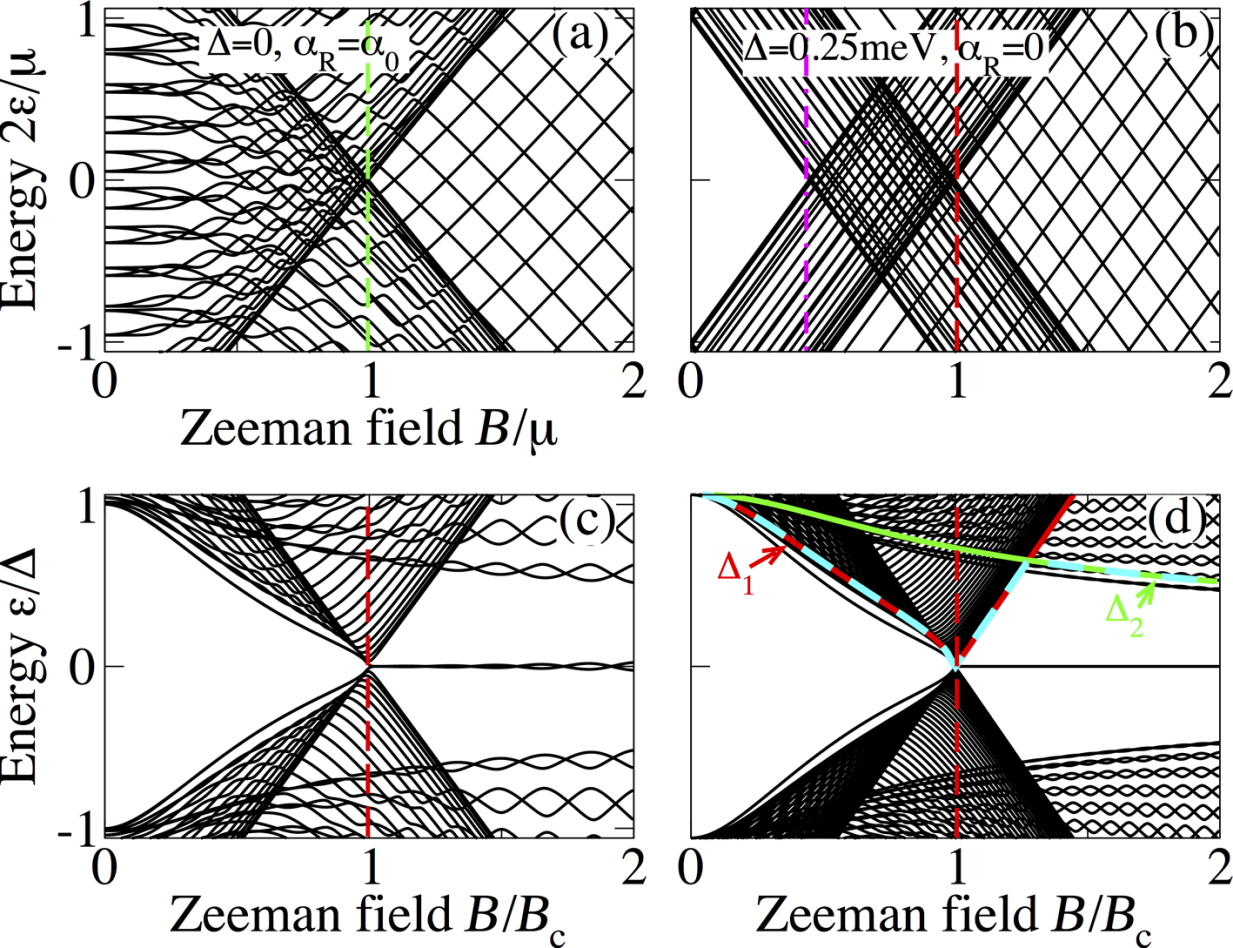
Low-energy spectrum of a superconducting nanowire as function of the Zeeman field *B*. At zero superconducting pairing with finite SOC the spectrum is gapless and becomes spin-polarized at *B* = μ as indicated by the green dashed line (a), while a finite superconducting pairing with zero SOC induces a gap for low values of *B* (b). As *B* increases, the induced gap is reduced and closed at *B* = Δ (vertical magenta dash-dot line). The bottom panels correspond to both finite superconducting pairing and SOC for *L*_S_ = 4000 nm (c) and *L*_S_ = 10000 nm (d). Note that as the Zeeman field increases the spectrum exhibits the closing of the gap at *B* = *B*_c_. While in the trivial phase, *B < B*_c_, there are no levels within the induced gap (c,d), in the topological phase for *B > B*_c_, the two lowest levels develop an oscillatory behaviour around zero energy (c). These lowest levels are the sought-for MBSs. For sufficiently long wires the amplitude of the oscillations is reduced (d) and these levels acquire zero energy. Solid red, green and dashed cyan curves indicate the induced gaps Δ*_1,2_* and min(Δ_1_, Δ_2_). Parameters: α_0_ = 20 meV·nm, μ = 0.5 meV, Δ = 0.25 meV and *L*_S_ = 4000 nm (a,b).

In Figure 2 we present the low-energy spectrum for a superconducting nanowire as a function of the Zeeman field at a fixed wire length *L*_S_. Figure 2a shows the case of zero superconducting pairing and finite SOC (Δ = 0, α_R_ ≠ 0), while Figure 2b shows a situation of finite pairing but with zero SOC (Δ ≠ 0, α_R_ = 0). These two extreme cases are very helpful in order to understand how a topological transition occurs when the missing ingredient (either superconducting pairing of finite SO) is included. This is illustrated in the bottom panels, which correspond to both finite SOC and superconducting pairing for *L*_S_ *<* 2ξ_M_ and *L*_S_ *>* 2ξ_M_, respectively. Here, ξ_M_ represents the Majorana localization length, which can be calculated from Equation 2[1,31],


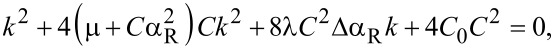


where 
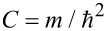
 and *C*_0_ = μ^2^ + Δ^2^ − *B*^2^. The Majorana localization length is defined as ξ_M_ = max[−1/k_sol_].

For the sake of the explanation, we plot the spectrum in the normal state (Δ = 0), Figure 2a, which is, of course, gapless. As the Zeeman field increases, the energy levels split and, within the weak Zeeman phase, *B <* μ, the spectrum contains energy levels with both spin components. In the strong Zeeman phase, *B >* μ, one spin sector is completely removed giving rise to a spin-polarized spectrum at low energies as one can indeed observe in Figure 2a. The transition point from weak to strong Zeeman phases is marked by the chemical potential *B* = μ (green dashed line). Figure 2b shows the low-energy spectrum at finite superconducting pairing, Δ ≠ 0, and zero SOC, α_R_ = 0. Firstly, we notice, in comparison with Figure 2a, that the superconducting pairing induces a gap with no levels for energies below Δ at *B* = 0, being in agreement with Anderson’s theorem [47]. A finite magnetic field induces a so-called Zeeman depairing, which results in a complete closing of the induced superconducting gap when *B* exceeds Δ. This is indeed observed in Figure 2b (magenta dash-dot line). Further increasing of the Zeeman field in this normal state gives rise to a region for Δ *< B < B*_c_, which depends on the finite value of the chemical potential (between red and magenta lines) where the energy levels contain both spin components (for μ = 0 the magenta dash-dot and the red dashed line coincide, not shown). Note that 
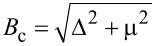
. For *B > B*_c_, one spin sector is removed and the energy levels are spin-polarized, giving rise to a set of Zeeman crossings that are not protected. Remarkably, when α_R_ ≠ 0, the low-energy spectrum undergoes a number of important changes, Figure 2c,d. First, the gap closing changes from Δ, Figure 2b, to 
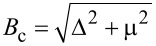
 (bottom panels). Second, a clear closing of the induced gap at *B* =*B*_c_ and reopening for *B > B*_c_ is observed as the Zeeman field increases. This can be seen by plotting the induced gaps Δ*_1,2_*, which are finite only at finite Zeeman fields. In Figure 2d, the red, green and dashed cyan curves correspond to Δ_1_, Δ_2_ and min(Δ_1_, Δ_2_). Remarkably, the closing and reopening of the induced gap in the spectrum follows exactly the gaps Δ*_1,2_* derived from the continuum (up to some finite-size corrections). Third, the spin-polarized energy spectrum shown in Figure 2b at zero SOC for *B > B*_c_ is washed out, keeping only the crossings around zero energy of the two lowest levels. This kind of closing and reopening of the spectrum at the critical field *B*_c_ indicates a topological transition where the two remaining lowest-energy levels for *B > B*_c_ are the well-known MBSs. Owing to the finite length *L*_S_, the MBSs exhibit the expected oscillatory behaviour due to their finite spatial overlap [48–51]. For sufficiently long wires 
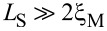
, the amplitude of the oscillations is considerably reduced (even negligible), which pins the MBSs to zero energy. Fourth, the SOC introduces a finite energy separation between the two lowest levels (crossings around zero) and the rest of the low-energy spectrum denoted here as “topological minigap”. Note that the value of this minigap, related to the high momentum gap Δ_2_, remains finite and roughly constant for strong SOC. In the case of weak SOC the minigap is reduced and for high Zeeman field it might acquire very small values, affecting the topological protection of the MBSs.

To complement this introductory part, calculations of the wavefunctions and charge density associated with the lowest levels of the topological superconducting nanowire spectrum are presented in the Supporting Information File 1.

## Results and Discussion

### Nanowire SNS junctions

**Figure 3:**
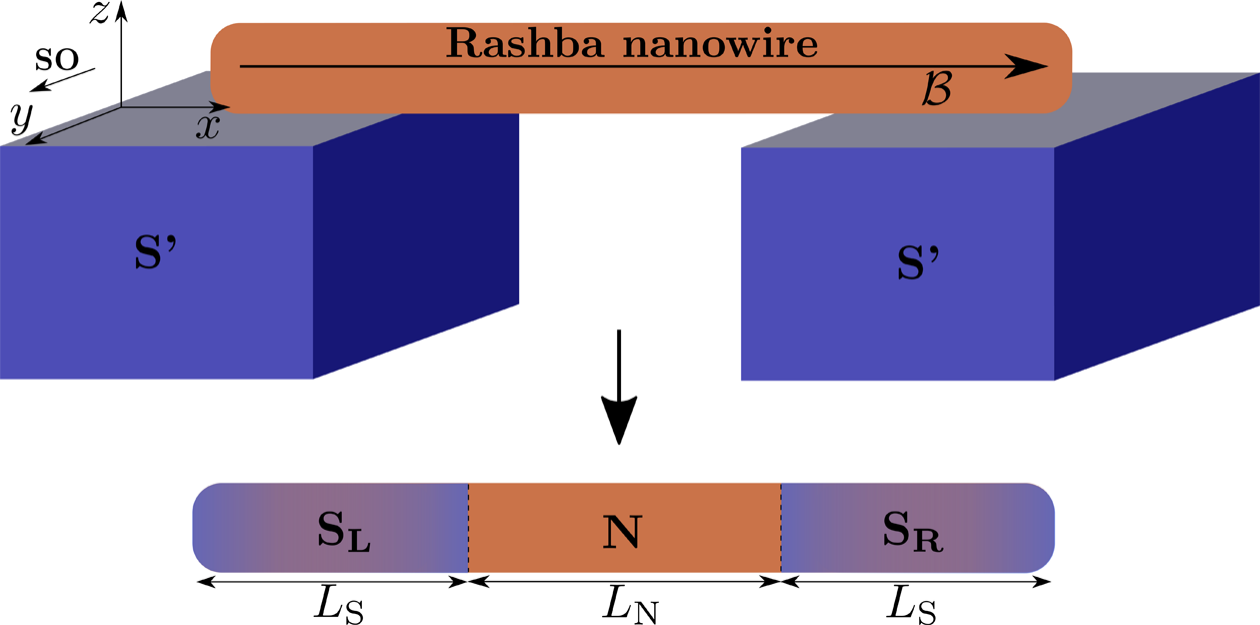
Schematic of SNS junctions based on Rashba nanowires. Top: A nanowire with Rashba SOC of length *L* = *L*_S_ + *L*_N_ + *L*_S_ placed on top of two *s*-wave superconductors (S’) with pairing potentials Δ_S′_ and subjected to an external magnetic field 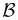 (denoted by the black arrow). Superconducting correlations are induced into the nanowire through the proximity effect. Bottom: Left and right regions of the nanowire become superconducting, denoted by *S*_L_ and *S*_R_, with induced pairing potentials 
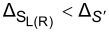
 and chemical potentials 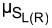, while the central region remains in the normal state with Δ_N_ = 0 and chemical potential μ_N_. This results in a superconductor–normal–superconductor (SNS) junction.

In this part, we concentrate on SNS junctions based on the proximitized nanowires that we discussed in the previous section. The basic geometry contains left (S_L_) and right (S_R_) superconducting regions of length *L*_S_ separated by a central normal (N) region of length *L*_N_, as shown in Figure 3. The regions N and S_L(R)_ are described by the tight-binding Hamiltonian *H*_0_ given by Equation 4 with their respective chemical potentials, μ_N_ and 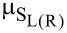. The Hamiltonian describing the SNS junction without superconductivity is then given by

[5]
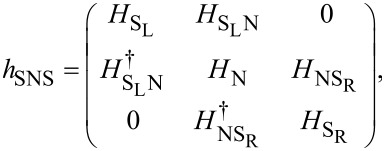


where 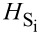 with i = L/R and *H*_N_ are the Hamiltonians of the superconducting and normal regions, respectively, 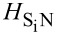 and 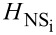 are the ones that couple *S*_i_ to the normal region *N*. The elements of these coupling matrices are non-zero only for adjacent sites that lie at the interfaces of the S regions and of the N region, while zero everywhere else. This coupling is parametrized between the interface sites by a hopping matrix *v*_0_ = τ*v*, where 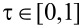, providing a good control of the normal transmission *T*_N_. The parameter τ controls the normal transmission that ranges from fully transparent (τ = 1) to tunnel (τ ≤ 0.6), as discussed in [37] for short junctions, being also valid for long junctions.

The superconducting regions of the nanowire are characterized by chemical potential 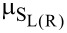 and the uniform superconducting pairing potentials [52–53] 
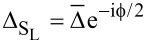
 and 
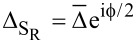
, where Δ *<* Δ_S′_ and 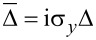. The central region of the nanowire is in the normal state without superconductivity, Δ_N_ = 0, and with chemical potential μ_N_. Thus, the pairing potential matrix in the junction space reads

[6]
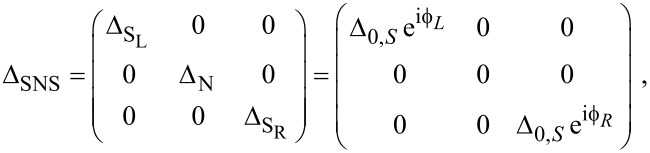


Next, we define the phase difference across the junction as 
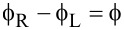
. Thus, the Hamiltonian for the full SNS junction reads in Nambu space [31,37]

[7]
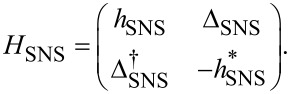


In what follows, we discuss short (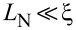) and long (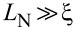) SNS junctions, where *L*_N_ is the length of the normal region and 
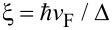
 is the superconducting coherence length [52]. The previous Hamiltonian is diagonalized numerically and in our calculations we consider realistic system parameters for InSb as described previously.

### Low-energy Andreev spectrum

**Figure 4:**
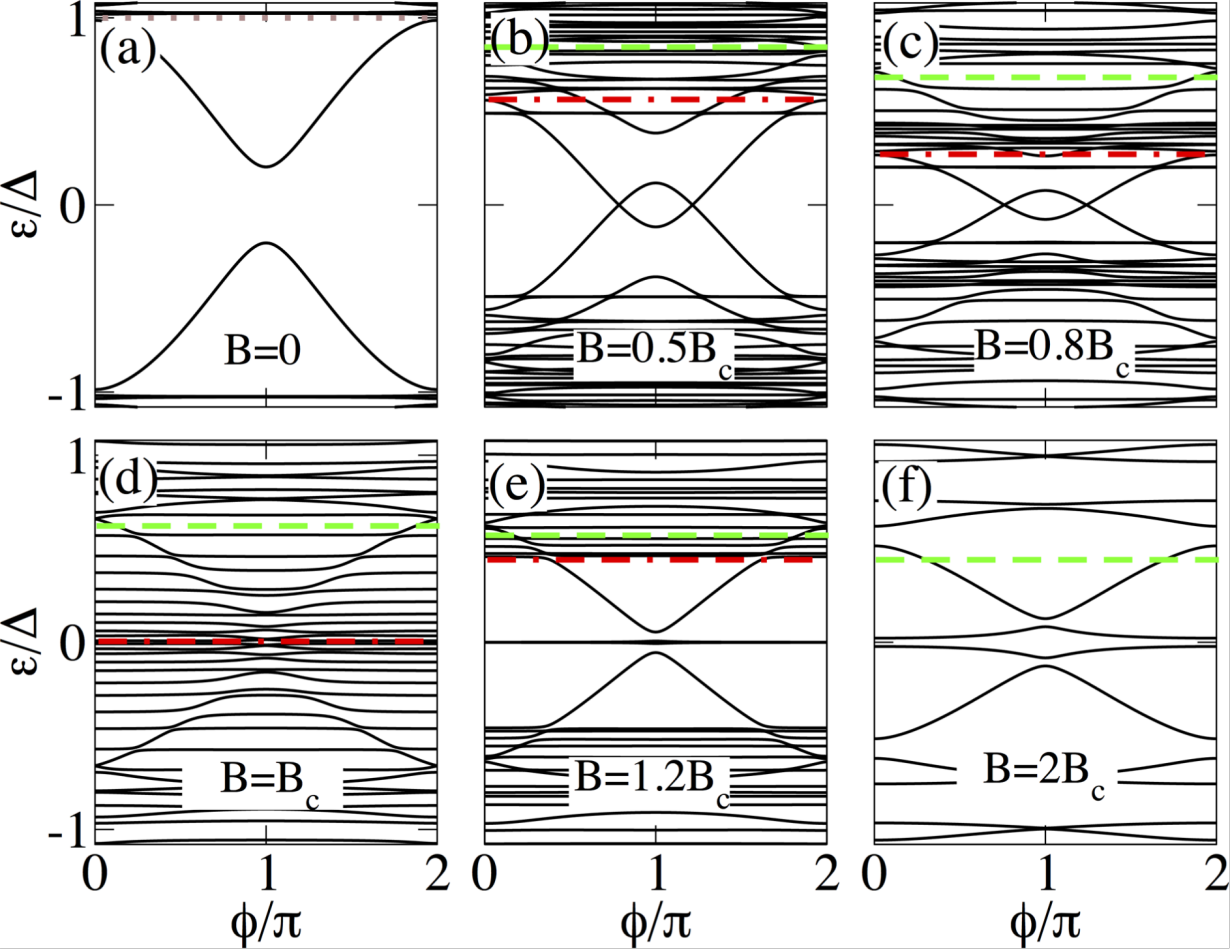
Low-energy Andreev spectrum as a function of the superconducting phase difference 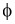 in a short SNS junction with *L*_N_ = 20 nm and *L*_S_ = 2000 nm. Different panels show the evolution with the Zeeman field: trivial phase for *B < B*_c_ (a–c), topological transition at *B* = *B*_c_ (d), and in the topological phase for *B > B*_c_ (e,f). The energy spectrum exhibits the two different gaps that appear in the system for finite Zeeman field (marked by red and green dashed horizontal lines). Note that after the gap inversion at *B* = *B*_c_, two MBSs emerge at the ends of the junction as almost dispersionless levels (outer MBSs), while two additional MBSs appear at 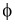 = π (inner MBSs). Parameters: α_R_ = 20 meV·nm, μ_N_ = μ_S_ = 0.5 meV and Δ = 0.25 meV.

**Figure 5:**
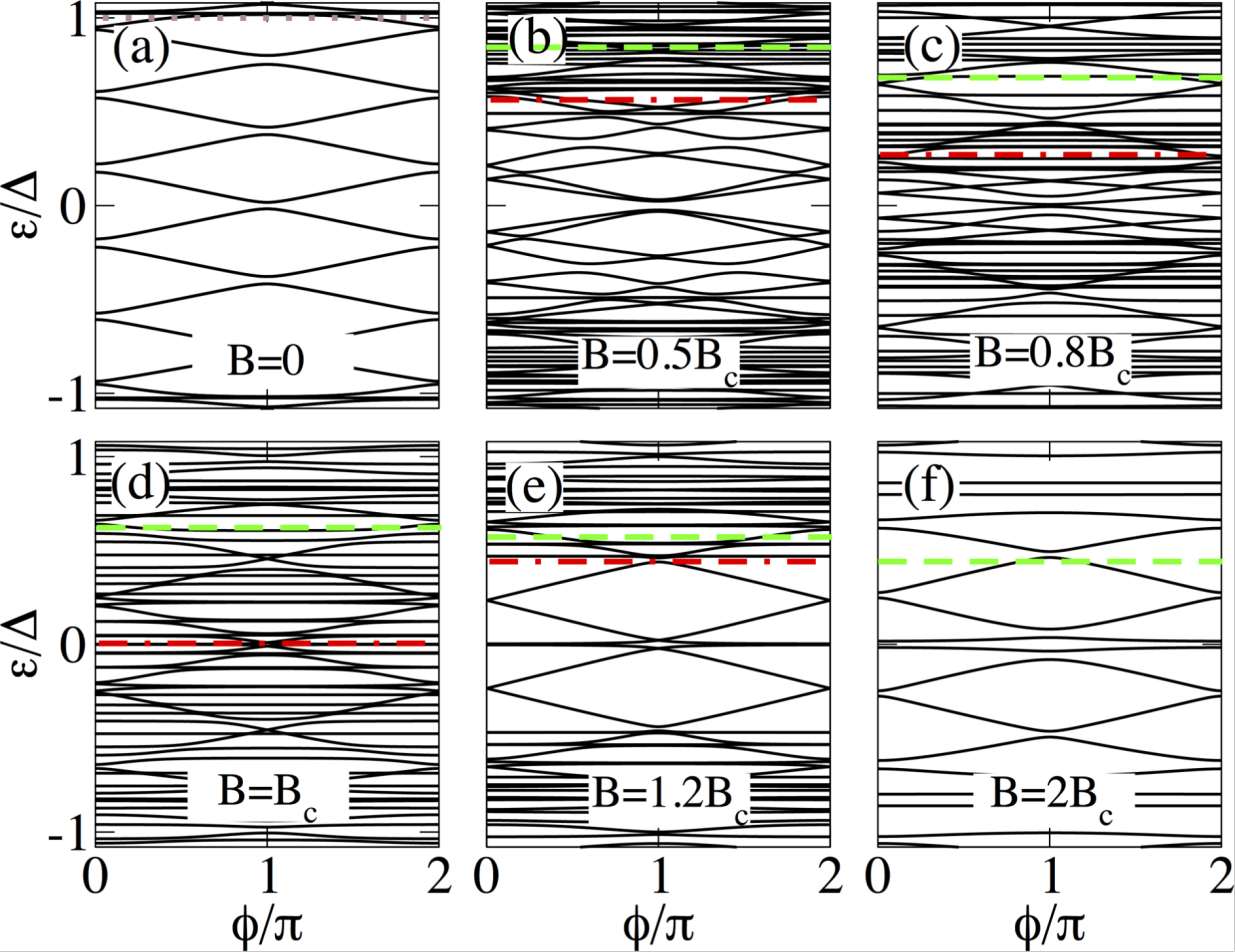
Same as in Figure 4 for a long junction with *L*_N_ = 2000 nm and *L*_S_ = 2000 nm. Note that, unlike short junctions, in this case the four lowest states for *B > B*_c_ coexist with additional levels within the induced gap which arise because *L*_N_ is longer.

**Figure 6:**
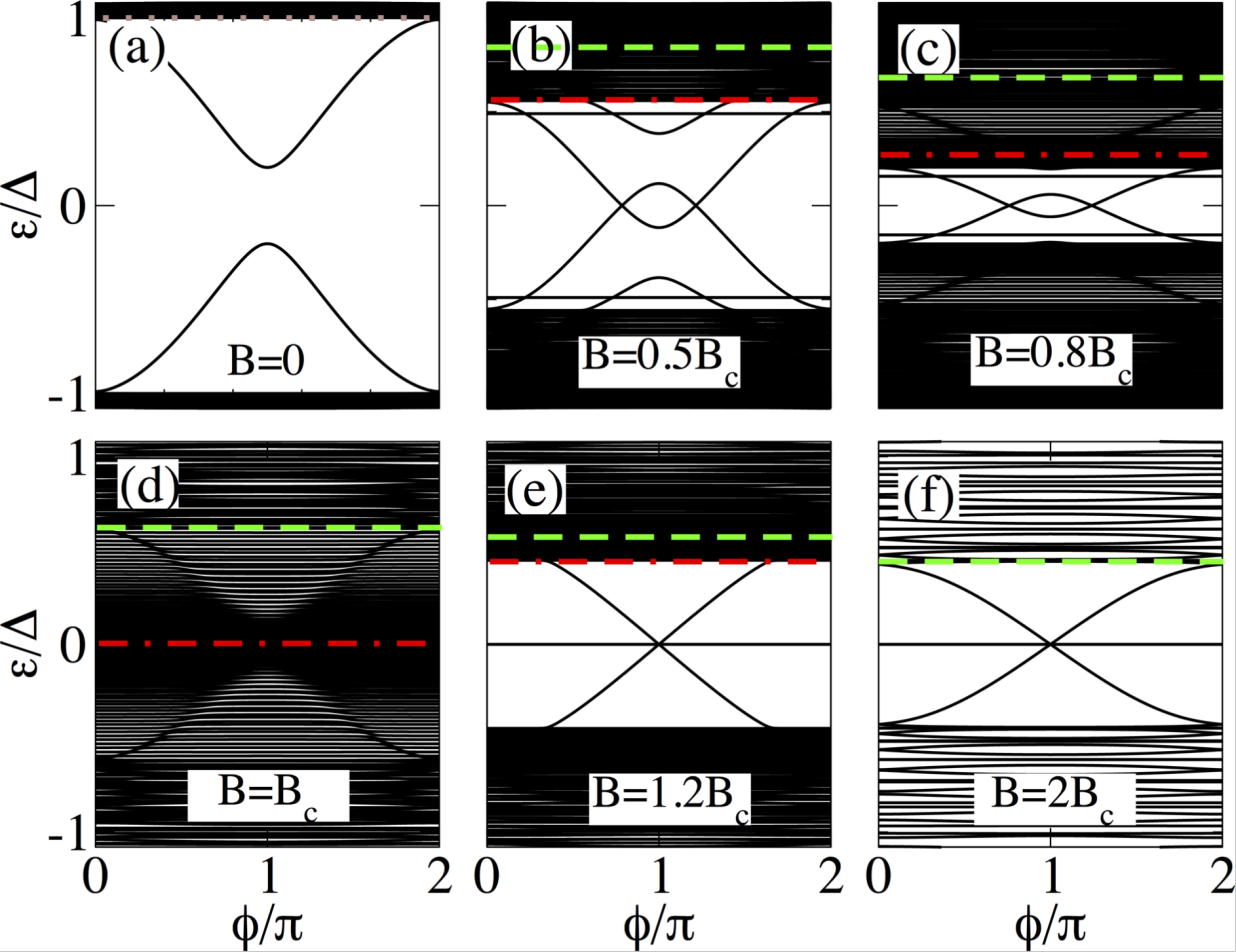
Same as in Figure 4 for a short junction with *L*_N_ = 20 nm and *L*_S_ = 10000 nm. Note that in this case, the emergent outer MBSs are dispersionless with 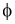, while the inner ones touch zero at 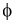 = π acquiring Majorana character.

**Figure 7:**
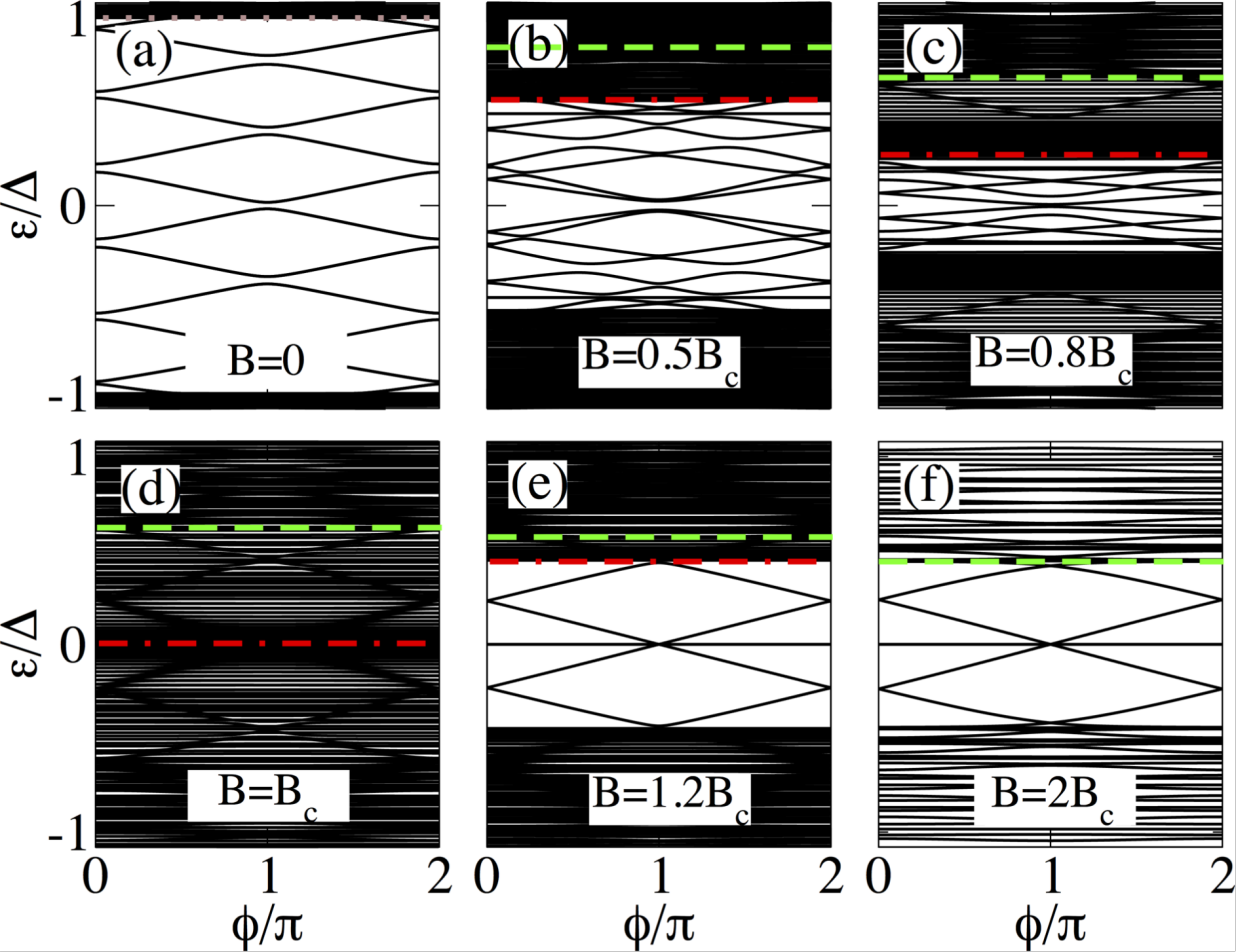
Same as in Figure 4 for a long junction with *L*_N_ = 2000 nm and *L*_S_ = 10000 nm. The four lowest levels coexist with additional levels. The outer MBSs lie at zero energy and the inner ones reach zero at 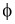 = π acquiring Majorana character.

Now, we are in a position to investigate the low-energy Andreev spectrum in short and long SNS junctions. In particular, we discuss the formation of Andreev bound states and their evolution from the trivial (*B < B*_c_) into the topological phases (*B > B*_c_). For this purpose we focus on the phase and the Zeeman-dependent low-energy spectrum in short and long junctions, presented in Figure 4 and Figure 5 for *L*_S_ ≤ 2ξ_M_. For completeness we also present the case of 
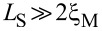
 in Figure 6 and Figure 7.

We first discuss short junctions with *L*_S_ ≤ 2ξ_M_. In this regime, at *B* = 0 two degenerate ABSs appear within Δ as solutions to the BdG equations described by Equation 7, see Figure 4a. It is interesting to point out that within standard theory for a transparent channel the ABS energies reach zero at 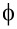 = π in the Andreev approximation 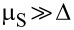 [54]. Figure 4a, however, shows that in general the ABS energies do not reach zero at 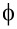 = π. The dense amount of levels above |ε*_p_*| *>* Δ represents the quasi-continuum of states, which consists of a discrete set of levels due to the finite length of the N and S regions. Moreover, it is worth to point out that the detachment (the space between the ABSs and quasi-continuum) of the quasi-continuum at 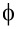 = 0 and 2π is not zero. It strongly depends on the finite length of the S regions (see Figure 6).

For a non-zero Zeeman field, Figure 4b and Figure 4c, the ABSs split and the two different gaps Δ_1_ and Δ_2_, discussed in section ‘Nanowire model’, emerge indicated by the dash-dot red and dashed green lines, respectively. By increasing the Zeeman field, the low-momentum gap Δ_1_ gets reduced (dash-dot red line), as expected, while the gap Δ_2_ (dashed green line) remains finite although it gets slightly reduced (Figure 4b and Figure 4c). For stronger, but unrealistic values of SOC we have checked that Δ_2_ is indeed constant. The two lowest levels in this regime, within Δ_1_ (dash-dot red line), develop a loop with two crossings that are independent of the length of the S region but exhibit a strong dependence on SOC, Zeeman field and chemical potential. We have checked that they appear due to the interplay of SOC and Zeeman field and disappear when μ acquires very large values, namely, in the Andreev approximation.

At *B* = *B*_c_, the energy spectrum exhibits the closing of the low-momentum gap Δ_1_, as indicated by red dash-dot line in Figure 4d. This indicates the topological phase transition, since two gapped topologically different phases can only be connected through a closing gap. By further increasing the Zeeman field, Figure 4e,f, *B > B*_c_, the inner gap Δ_1_ acquires a finite value again. This reopening of Δ_1_ indicates that the system enters into the topological phase and the superconducting regions denoted by S_L(R)_ become topological, while the *N* region remains in the normal state. Thus, MBSs are expected to appear for *B > B*_c_ at the ends of the two topological superconducting sectors, since they define interfaces between topologically different regions.

This is what we indeed observe for *B > B*_c_ in Figure 4e and Figure 4f, where the low-energy spectrum has Majorana properties. In fact, for *B > B*_c_, the topological phase is characterized by the emergence of two (almost) dispersionless levels with 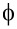, which represent the outer MBSs γ*_1,4_* formed at the ends of the junction. Additionally, the next two energy levels strongly depend on 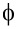 and tend towards zero at 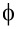 = π, representing the inner MBSs γ*_2,3_* formed inside the junction. For sufficiently strong fields, *B* = 2*B*_c_, the lowest gap is Δ_2_ indicated by the green dashed line, which in principle bounds the MBSs. The quasi-continuum in this case corresponds to the discrete spectrum above and below Δ_2_, where Δ_2_ is the high-momentum gap marked by the green dashed horizontal line in Figure 4e,f.

The four MBSs develop a large splitting around 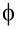 = π, which arises when the wave-functions of the MBSs have a finite spatial overlap *L*_S_ ≤ 2ξ_M_. Since the avoided crossing between the dispersionless levels (belonging to γ*_1,4_*) and the dispersive levels (belonging to γ*_2,3_*) around 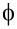 = π depends on the overlap of MBSs on each topological segment. It can be used to quantify the degree of Majorana non-locality (a variant of this idea using quantum-dot parity crossings has been discussed in [55–56]). This can be explicitly checked by considering SNS junctions with longer superconducting regions, where the condition 
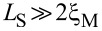
 is fulfilled such that the energy splitting at 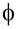 = π is reduced.

As an example, we present in Figure 6 the energy levels as a function of the phase difference for 
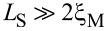
, where the low-energy spectrum undergoes some important changes. First, we notice in Figure 6 that the energy spectrum at *B* = 0 for |ε*_p_*| *>* Δ, exhibits a visibly denser spectrum than that in Figure 4 signaling the quasi-continuum of states. Notice that in the topological phase, *B > B*_c_, the lowest two energy levels, associated to the outer MBSs, are insensitive to 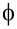 remaining at zero energy. Thus, they can be considered as truly zero modes. On the other hand, the inner MBSs are truly bound within Δ_2_, unlike in Figure 4, and for 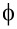 = 0 and 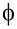 = 2π they merge with the quasi-continuum at ±Δ. Thus, an increase in the length of the superconducting regions favors the reduction of the detachment between the discrete spectrum and the quasi-continuum at 0 and 2π, as it should be for a ballistic junction [23–24]. Moreover, the energy splitting at 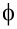 = π is considerably reduced, even negligible. However, it will be always non-zero, though not visible to the naked eye, due to the finite length and, thus, due to the presence of the outer MBSs.

The low-energy spectrum as a function of the superconducting phase difference for different values of the Zeeman field in long SNS junctions is presented in Figure 5 for *L*_S_ ≤ 2ξ_M_. Additionally, we show in Figure 7 the case for 
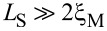
.

As expected, long junctions contain more levels within the energy gap Δ, see Figure 5a and Figure 7a, than short junctions. As we shall discuss, this eventually affects the signatures of Majorana origin in the supercurrents for *B B*_c_, namely, the ones corresponding to the lowest four levels.

**Figure 8:**
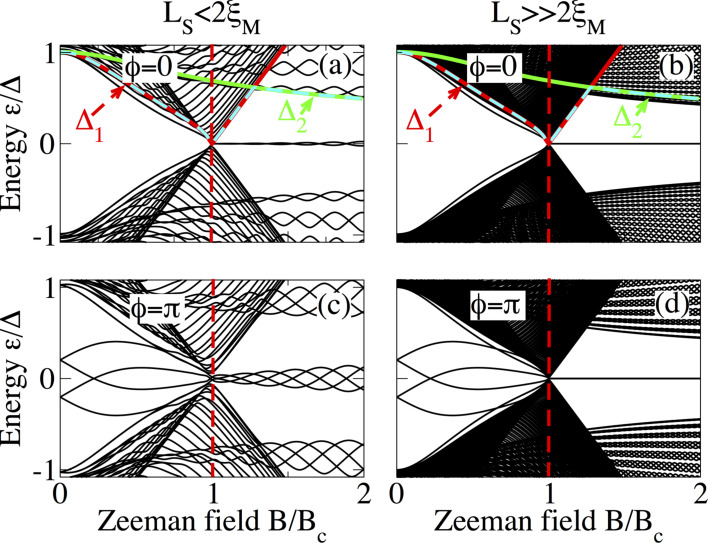
Low-energy Andreev spectrum as a function of the Zeeman field in a short SNS junction at 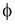 = 0 (a,b) and 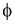 = π (c,d) with *L*_S_ = 2000 nm (a,c) and *L*_S_ = 10000 nm (b,d). The low-energy spectrum traces the gap closing and reopening by the solid red curve that corresponds to Δ_1_, while for *B > B*_c_ we observe the emergence of two MBSs at 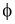 = 0 (a) and four MBSs at 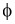 = π (c), which oscillate around zero energy with *B* due to *L*_S_ ≤ 2ξ_M_ within a minigap defined by Δ_2_ (solid green curve). For 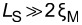 the MBSs are truly zero modes (b,d). Parameters: *L*_N_ = 20 nm, α_R_ = 20 meV·nm, μ = 0.5 meV and Δ = 0.25 meV.

**Figure 9:**
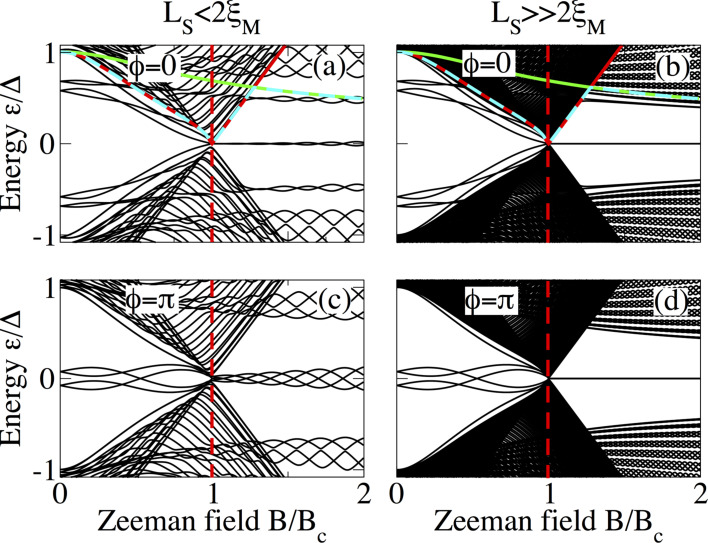
Same as in Figure 8 for an intermediate junction with *L*_N_ = 400 nm.

**Figure 10:**
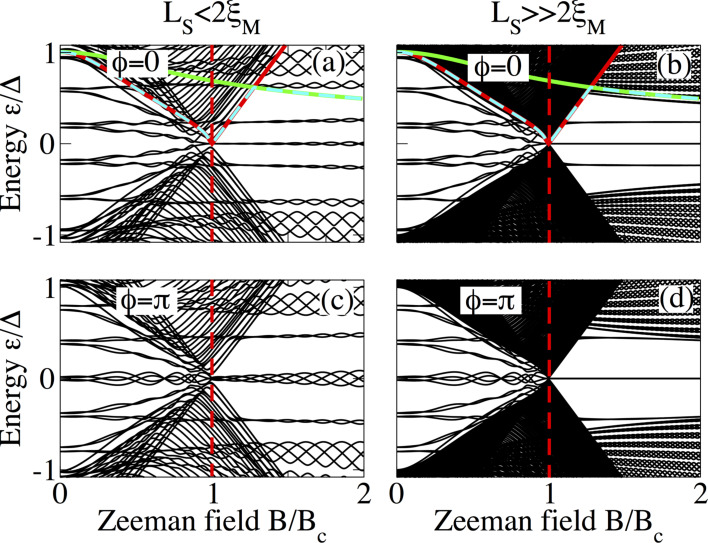
Same as in Figure 8 for a long junction with *L*_N_ = 2000 nm.

The above discussion can be clarified by considering the dependence of the low-energy spectrum on the Zeeman field at fixed phase difference 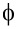 = 0 and 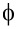 = π. This is shown in Figure 8 (short junction limit), Figure 9 (intermediate junction limit) and Figure 10 (long junction limit) for *L*_S_ ≤ 2ξ_M_ (panels a and c in each figure) and 
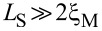
 (panels b and d in each figure). In panels a and b, the gaps Δ_1_, Δ_2_ and min(Δ_1_,Δ_2_) are also plotted as solid red, solid green and dashed cyan lines to visualize the gap closing and reopening discussed in the previous section. In all cases, it is clear that MBS smoothly evolve from the lowest ABS either following the closing of the induced gap Δ_1_, indicated by the solid red curve, at 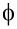 = 0 or evolving from the lowest detached levels at 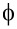 = π. The latter first cross zero energy, owing to Zeeman splitting, and eventually become four low-energy levels oscillating out of phase around zero energy (Figure 8c). The emergence of these oscillatory low-energy levels, separated by a minigap Δ_2_, indicated by the solid green curve, from the quasi-continuum characterizes the topological phase of the SNS junction. As expected, the oscillations become reduced for 
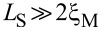
 and the four levels at 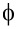 = π become degenerate at zero energy, see Figure 8b,d.

An increase in the length of the normal section results in an increase of the amount of subgap levels as observed in Figure 9 and Figure 10. In both cases, in the topological phase, *B > B*_c_, these additional levels reduce the minigap with respect to short junctions and also slightly reduce the amplitude of the oscillations of the energy levels around zero as seen Figure 9a and Figure 9b as well as Figure 10a and Figure 10b. Also, the minigaps for 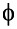 = 0 and 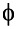 = π are different, in contrast to short junctions. In fact, the minigap at 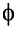 = 0 is almost half of the value at 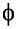 = π for the chosen parameters. This can be understood from the phase dispersion of the long junction ABS spectra such as the ones in Figure 5 and Figure 7. For longer N regions, this difference can be even larger.

From the above discussion it is clear that the energy spectrum of SNS nanowire junctions offers the possibility to directly monitor the ABSs that trace the gap inversion and eventually evolve into MBSs.

### Supercurrents

After having established in detail the energy spectrum in short and long SNS junctions, we now turn our attention to the corresponding phase-dependent supercurrents. They can be calculated directly from the discrete Andreev spectrum ε*_p_* as [37,54,57]:

[8]
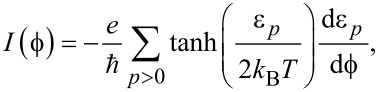


where *k_rmB_* is the Boltzmann constant, *T* is the temperature and the summation is performed over positive eigenvalues ε*_p_*. By construction, our junctions have finite length, which implies that Equation 8 exactly includes the discrete quasi-continuum contribution.

**Figure 11:**
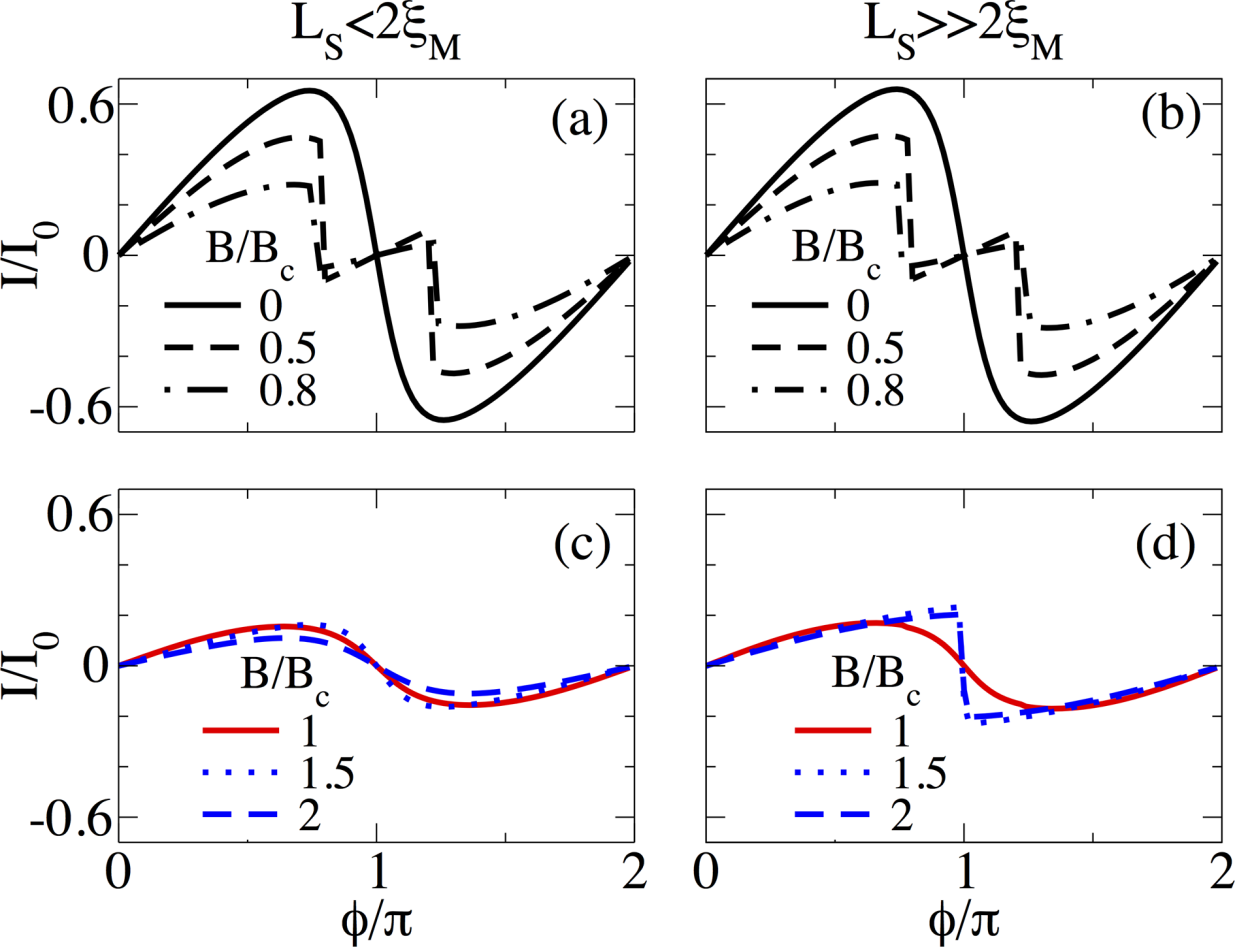
Supercurrent as a function of the superconducting phase difference in a short SNS junction, *I*(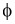), for *L*_S_ = 2000 nm ≤ 2ξ_M_ (a,c) and *L*_S_ = 10000 nm 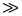 2ξ_M_ (b,d). Panels a and b show the Josephson current in the trivial phase for different values of the Zeeman field, *B < B*_c_, while panels c and d correspond to different values of the Zeeman field in the topological phase, *B* ≥ *B*_c_. Note the sawtooth feature at 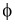 = π for 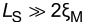. Parameters: α_R_=20 meV·nm, μ = 0.5 meV, Δ = 0.25 meV and 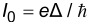.

**Figure 12:**
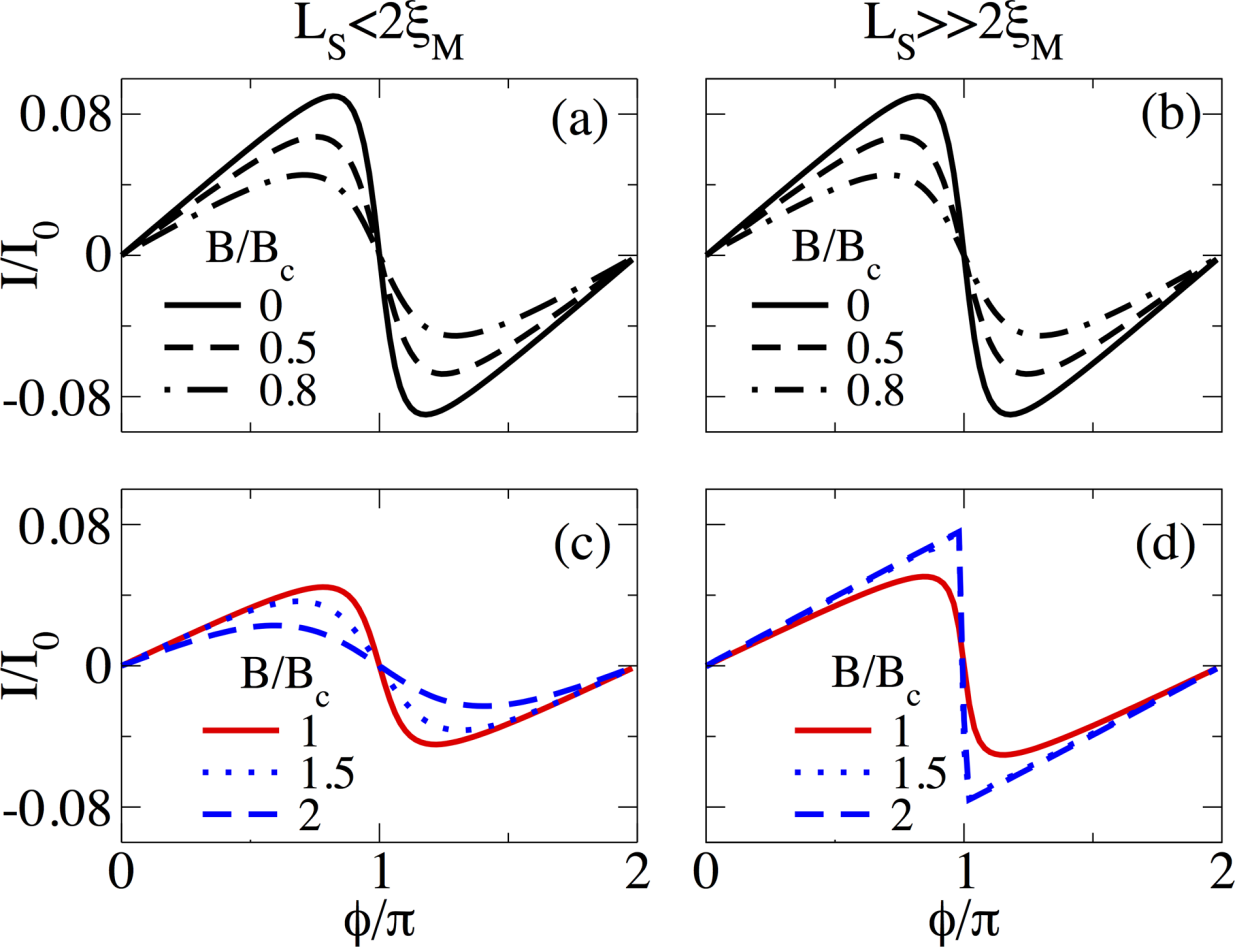
Supercurrent as a function of the superconducting phase difference in a long SNS junction with *L*_N_ = 2000 nm, for *L*_S_ = 2000 nm ≤ 2ξ_M_ (a,c) and *L*_S_ = 10000 nm 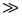 2ξ_M_ (b,d). Panels a and b show the Josephson current in the trivial phase for different values of the Zeeman field, *B < B*_c_, while panels c and d correspond to different values of the Zeeman field in the topological phase, *B* ≥ *B*_c_. In this case the magnitude of the supercurrent is reduced, an effect caused by the length of the normal section.

In Figure 11 and Figure 12 we present supercurrents as a function of the superconducting phase difference *I*(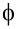) for different values of the Zeeman field in short and long SNS junctions, respectively. Panels a and c correspond to *L*_S_ ≤ 2ξ_M_, while panels b and d correspond to 
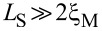
.

First, we discuss the short junction regime presented in Figure 11. At *B* = 0 the supercurrent *I*(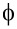) has a sine-like dependence on 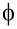, with a relative fast change of sign around 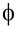 = π that is determined by the derivative of the lowest-energy spectrum profile around 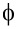 = π. This result is different from usual ballistic full transparent supercurrents in trivial SNS junctions [54], where the supercurrent is proportional to sin(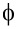/2) being maximum at 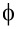 = π. This difference from the standard ballistic limit can be ascribed to deviations from the ideal Andreev approximation, see also the discussion of Figure 4a, owing to the relatively low chemical potential needed to reach the helical limit and, eventually, the topological regime as the Zeeman field increases. At finite values of the Zeeman field *B*, but still in the trivial phase *B < B*_c_ (dashed and dash-dot curves), *I*(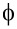) undergoes changes. First, the maximum value of *I*(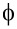) is reduced due to the reduction of the induced gap that is caused by the Zeeman effect. Second, *I*(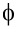) develops a zig-zag profile (just before and after 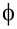 = π) signalling a 0–π transition in the supercurrent. This transition arises from the zero-energy crossings in the low-energy spectrum, see Figure 4b,c. As discussed above, these level crossings result from the combined action of both SOC and Zeeman field at low μ, and introduce discontinuities in the derivatives with respect to 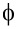. Besides these features, all the supercurrent curves for *B < B*_c_, for both *L*_S_ ≤ 2ξ_M_ and 
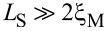
, exhibit a similar behavior, see Figure 11. Interestingly, the system is gapless at the topological transition, *B* = *B*_c_, but the supercurrent remains finite, see red curve in Figure 11c.

For *B > B*_c_, the S regions of the SNS junction become topological and MBSs emerge at their ends, as described in the previous subsection. Despite the presence of MBSs, the supercurrent *I*(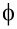) remains 2π-periodic, i.e., *I*(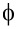) = *I*(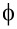 + 2π). This results from the fact that we sum up positive levels only, as we deal with an equilibrium situation. Since the supercurrent is a ground state property, transitions between the negative and positive energies are not allowed, because of an energy gap originating from Majorana overlaps. Strategies to detect the presence of MBSs beyond the equilibrium supercurrents described here include the ac Josephson effect, noise measurements, switching-current measurements, microwave spectroscopy and dynamical susceptibility measurements [25–30].

As the Zeeman field is further increased in the topological phase, *B > B*_c_, the supercurrent tends to decrease due to the finite Majorana overlaps when *L*_S_ ≤ 2ξ_M_, see dotted and dashed blue curves in Figure 11d. On the other hand, as the length of S becomes larger such that 
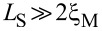
 the overlap is reduced, which is reflected in a clear sawtooth profile at 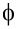 = π, see dotted and dashed blue curves in Figure 11d. This discontinuity in *I*(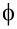) depends on *L*_S_ and results from the profile of the lowest-energy spectrum at 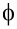 = π, as shown in Figure 6d. The sawtooth profile thus indicates the emergence of well-localized MBSs at the ends of S and represents one of our main findings.

As discussed above, the supercurrent for *B < B*_c_, Figure 11a and Figure 11b, does not depend on *L*_S_. In contrast, supercurrents in the topological phase, Figure 11c and Figure 11d, do strongly depend on *L*_S_ owing to the emergence of MBSs.

In Figure 12 we present *I*(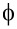) for long junctions 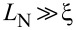 at different values of the Zeeman field. Panels a and c correspond to *L*_S_ ≤ 2ξ_M_ and panels b and d correspond to 
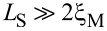
. Even though the maximum value of the current is reduced in this regime, the overall behavior is very similar to the short-junction regime for both *B < B*_c_ and *B > B*_c_. The only important difference with respect to the short junction case is that *I*(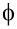) in the long-junction regime does not exhibit the zig-zag profile due to 0–π transitions.

As the system enters into the topological phase for *B > B*_c_ and *L*_S_ ≤ 2ξ_M_, Figure 12c, the maximum supercurrent decreases, indicating the non-zero splitting at 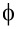 = π in the low-energy spectrum. Deep in the topological phase, the supercurrent exhibits a slow (slower than in the trivial phase Figure 12a) sign change around 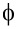 = π, and its dependence on 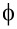 tends to approach a sine function. However, for 
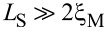
, shown in Figure 12d, the supercurrent acquires an almost constant value as *B* increases and develops a clear sawtooth profile at 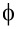 = π due to the zero energy splitting in the low-energy spectrum at 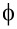 = π, similarly to the short-junction case. It is worth to point out that, although the maximum supercurrent is slightly reduced, deep in the topological phase (dashed and dotted blue curves) its maximum value is approximately close to the maximum value in the trivial phase, which is different from what we found in the short-junction case. This is a clear consequence of the emergence of additional levels within the induced gap due to the increase of *L*_N_. These additional levels exhibit a strong dependence on the superconducting phase, very similar to the inner MBSs as one can see in Figure 5e,f.

**Figure 13:**
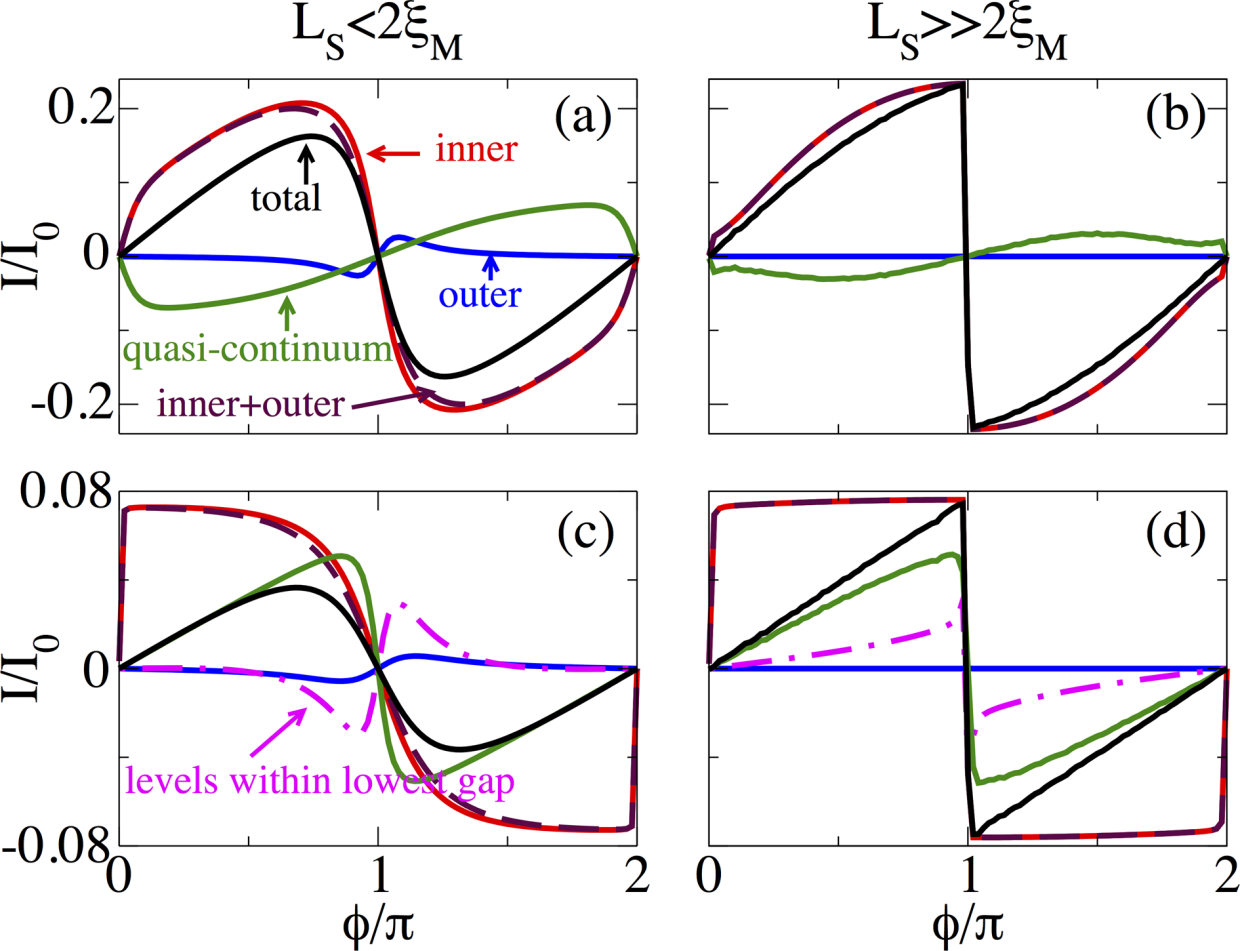
Supercurrent as a function of 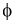 at *B* = 1.5*B*_c_. Contributions to the supercurrent for (a,b) short and (c,d) long junctions. (a,c) *L*_S_ ≤ 2ξ_M_ and (b,d) 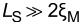. The different curves in (a,b) correspond to individual contributions to *I*(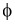) from outer, inner, and outer + inner (levels within the lowest induced gap Δ_2_), quasi-continuum (levels above the lowest gap Δ_2_) and total levels. In (c,d), the additional magenta curve corresponds to all levels within Δ_2_. In long junctions the number of levels within Δ_2_ exceeds the number of MBSs. MBSs coexist with additional levels within Δ_2_. Parameters: α_R_ = 20 meV·nm, μ = 0.5 meV, Δ = 0.25 meV and 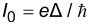.

In order analyze the individual contribution of outer and inner MBSs with respect to the quasi-continuum we calculate and identify supercurrents for such situations. This is presented in Figure 13 for short junctions (Figure 13a,b) and for long junctions (Figure 13c,d). In this regime the lowest gap is the high-momentum gap Δ_2_, and the levels outside this gap constitute the quasi-continuum.

Firstly, we discuss short junctions. The supercurrent due to outer MBSs for *L*_S_ ≤ 2ξ_M_ is finite only around 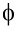 = π, exhibiting an odd dependence on 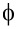 around π. However, away from this point it is approximately zero and independent of 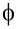 (see blue curve in Figure 13a). When 
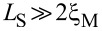
, the outer MBSs are very far apart and their contribution to *I*(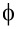) is zero (see blue curve in Figure 13b). On the other hand, the contribution of the inner MBSs to *I*(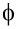) is enormous and the outer part only slightly changes the shape of the maximum supercurrent when *L*_S_ ≤ 2ξ_M_, while for 
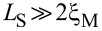
 the outer MBSs do not contribute, as shown by the dashed brown curve in Figure 13a,b. Moreover, the inner contribution exhibits roughly the same dependence on 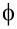 as the contribution of the whole energy spectrum shown by the black curve in Figure 13a,b. As described in the previous subsection, the quasi-continuum is considered to be the discrete energy spectrum above |ε*_p_*| *>* Δ_2_. The quasi-continuum contribution to *I*(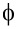) is finite and odd in 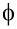 around π, as shown by green curves in Figure 13a,b. The quasi-continuum contribution to the total supercurrent *I*(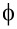) far away from 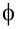 = π is appreciable mainly when the MBSs exhibit a finite energy splitting as seen in Figure 13a. Interestingly, the outer and in particular the inner MBSs (levels within Δ_2_) are the main source when such overlap is completely reduced and determine the profile of *I*(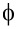), as shown in Figure 13b.

In long junctions the situation is different, mainly because more levels emerge within Δ in the trivial phase. For *B > B*_c_ within Δ_2_ these additional levels coexist with the inner and outer MBSs, see Figure 13c,d. The contribution of the outer MBSs to *I*(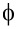) exhibits roughly similar behaviour as for short junctions although smoother around 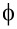 = π , shown by the blue curve in Figure 13c,d. The inner MBSs, however, have a strong dependence on 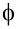 and develop their maximum value close to 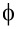 = 2π*n* with *n* = 0,1,… (see red curve). The outer MBSs almost do not affect the overall shape of the *I*(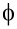) curve (see dashed brown curve). Since a long junction hosts more levels, we also show by the dash-dot magenta curve the contribution of all the levels within Δ_2_, including also the four MBSs. This contribution is considerably large only close to 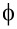 = π, with a minimum and maximum value before and after 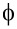 = π for *L*_S_ ≤ 2ξ_M_, respectively. This is indeed the reason why the supercurrent is reduced as *B* increases in the topological phase for *L*_S_ ≤ 2ξ_M_, see dotted and dashed blue curves in Figure 12c. For 
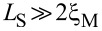
 the contribution of all the levels within Δ_2_ exhibits a sawtooth profile at 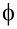 = π, which, instead of reducing the quasi-continuum contribution (green curve), increases the maximum value of *I*(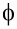) at 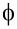 = π resulting in the solid black curve. Importantly, unlike in short junctions, in long junctions the quasi-continuum modifies *I*(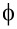) around 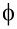 = π. Thus, a zero-temperature current-phase measurement in an SNS junction setup could indeed reveal the presence of MBSs by observing the reduction of the maximum supercurrent. In particular, well-localized MBSs are revealed in the sawtooth profile of *I*(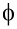) at 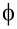 = π. In what follows we analyze the effect of temperature, variation of normal transmission and random disorder on the sawtooth profile at 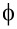 = π of the supercurrent.

#### Temperature effects

**Figure 14:**
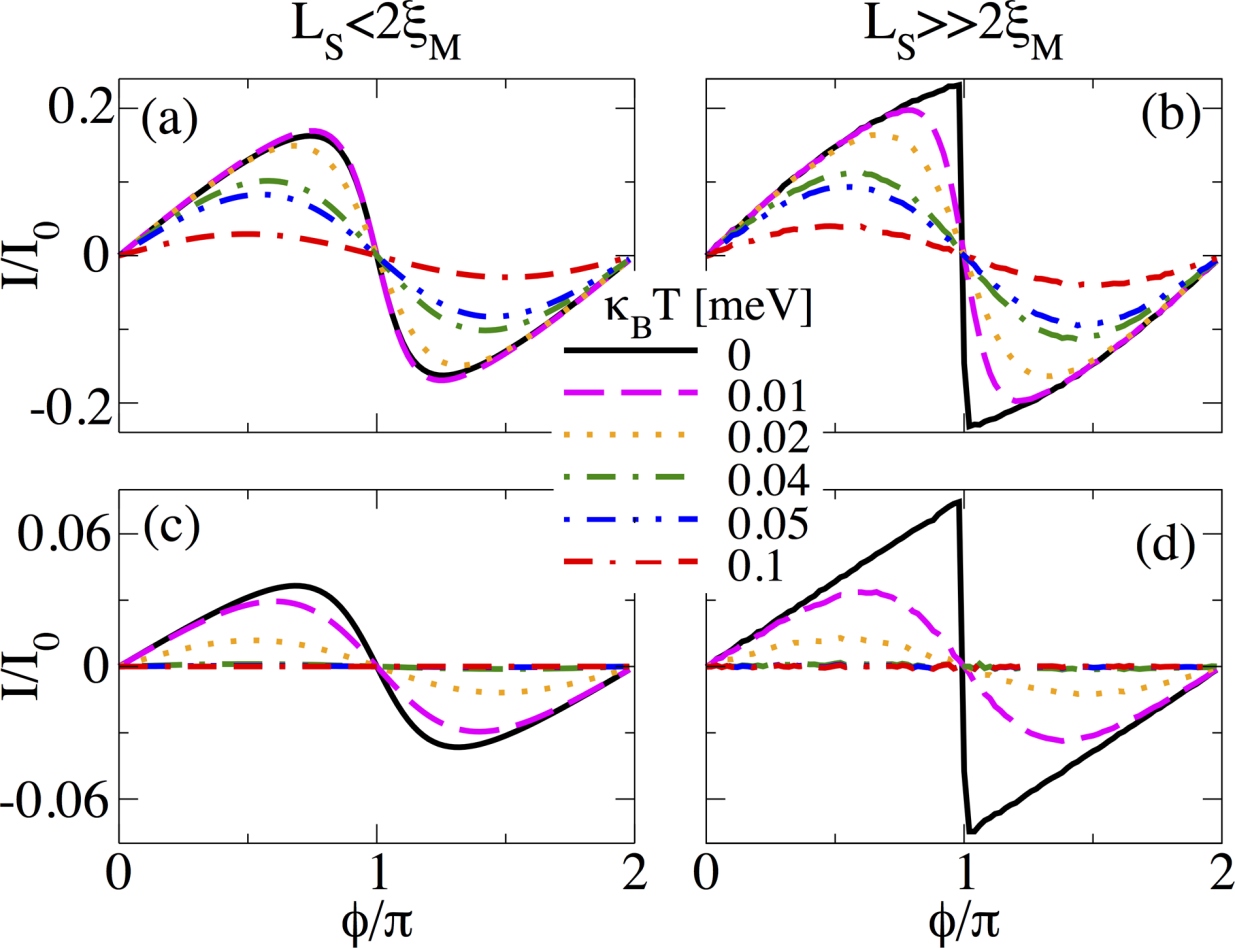
Finite temperature effect on the supercurrent, *I*(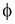), in (a,b) a short and (c,d) a long junction. (a,c) *L*_S_ = 2000 nm ≤ 2ξ_M_ and (b,d) *L*_S_ = 10000 nm 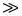 2ξ_M_. Different curves correspond to different values of *k*_B_*T*. The sawtooth profile smooths out at finite temperature. Parameters: *L*_N_ = 20 nm for short and *L*_N_ = 2000 nm for long junctions, α_R_ = 20 meV·nm, μ = 0.5 meV, Δ = 0.25 meV and 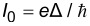.

In this part, we analyze the effect of temperature on supercurrents in the topological phase. In Figure 14 we present the supercurrent as a function of the superconducting phase difference, *I*(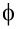), in the topological phase *B* = 1.5*B*_c_ at different temperature values for *L*_S_ ≤ 2ξ_M_ (Figure 14a) and 
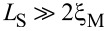
 (Figure 14b). At zero temperature, for *L*_S_ ≤ 2ξ_M_, shown by the black solid curve in Figure 14a, the dependence of the supercurrent on 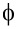 approximately corresponds to a sine-like function. A small increase in temperature *k*_B_*T* = 0.01 meV (magenta dashed curve) slightly modifies the profile of the maximum supercurrent. However, for 
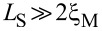
 (Figure 14b), the same temperature (dashed curve) value has a detrimental effect on the sawtooth profile of *I*(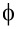) at 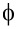 = π, which reduces the maximum value and smooths the curve out due to the thermal population of ABSs. We have checked that smaller temperature values than the ones presented in Figure 14 also smooth out the sawtooth profile but the fast sign change around 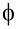 = π is still visible. This effect remains as long as 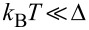. As the temperature increases, *I*(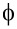) smoothly acquires a true sine shape, as seen in Figure 14a. Although the sawtooth profile might be hard to observe in real experiments, the maximum value of *I*(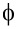), which is finite in the topological phase and almost halved with respect to the trivial phase in short junctions [37], still provides a measure to distinguish it from *I*(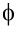) in trivial junctions.

#### Normal transmission effects

The assumption of perfect coupling between N and S regions in previous calculations is indeed a good approximation because of the enormous advances in fabrication of hybrid systems. However, it is also relevant to study whether the sawtooth profile of *I*(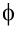) is preserved or not when the normal transmission *T*_N_, described by τ, is varied.

**Figure 15:**
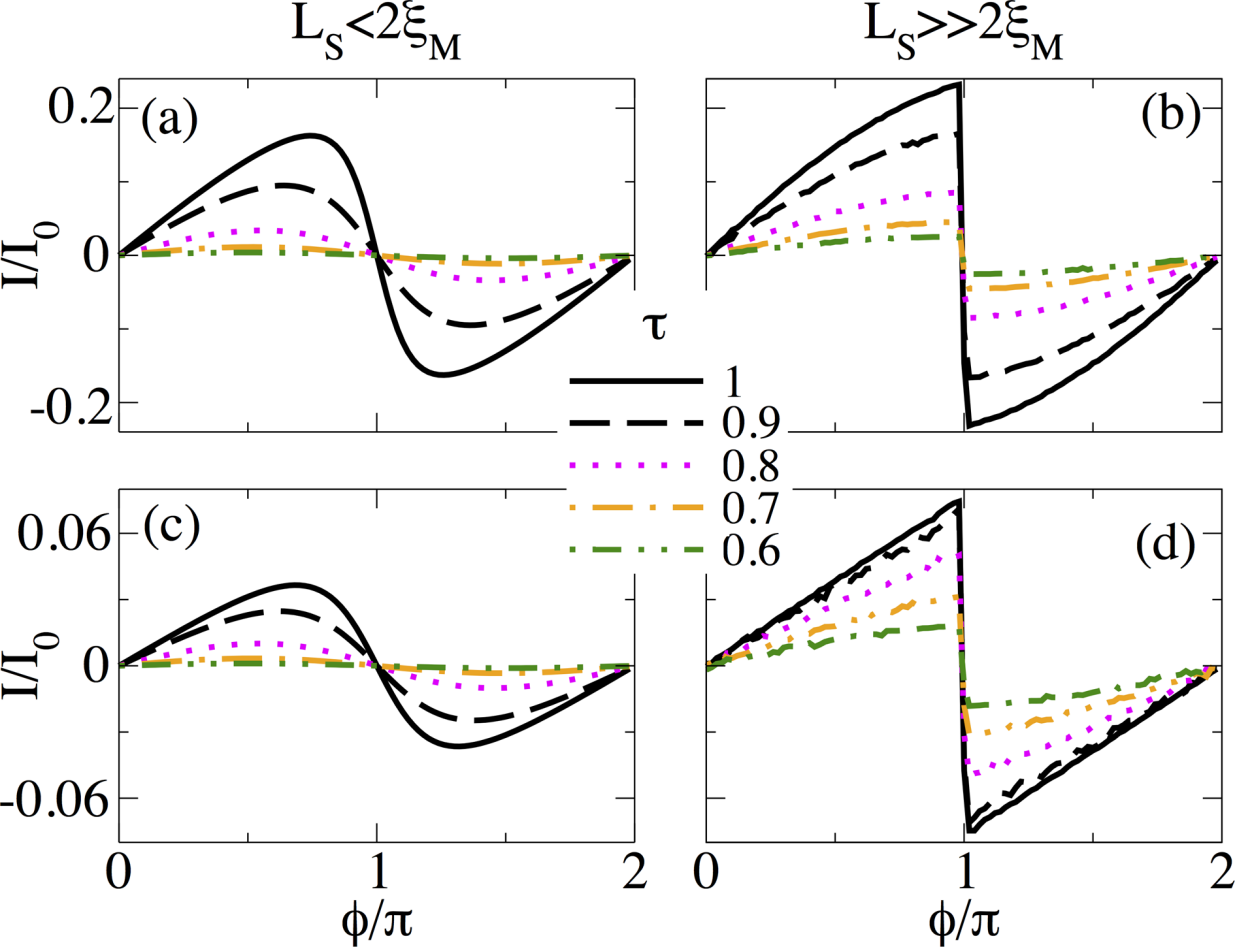
Effect of normal transmission through the coupling parameter τ on the supercurrent, *I*(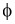), in (a,b) a short and (c,d) a long SNS junction. (a,c) *L*_S_ = 2000 nm ≤ 2ξ_M_ and (b,d) *L*_S_ = 10000 nm 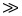 2ξ_M_. Although after decreasing τ the magnitude of the supercurrent at 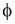 = π decreases, the sawtooth profile is preserved. Parameters: *L*_N_ = 20 nm for short and *L*_N_ = 2000 nm for long junctions, α_R_ = 20meV·nm, μ = 0.5 meV, Δ = 0.25 meV and 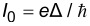.

Figure 15 shows the supercurrent *I*(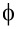) in short junctions at *B* = 1.5*B*_c_ for different values of τ for *L*_S_ ≤ 2ξ_M_ (Figure 15a) and 
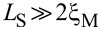
 (Figure 15b). When τ is reduced, the supercurrent *I*(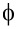) is also reduced. However, for *L*_S_ ≤ 2ξ_M_, there is a transition from a sudden sign change around 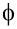 = π to a true sine function with reducing τ, very similar to the effect of temperature discussed above. Notice that in the tunnel regime, τ = 0.6, *I*(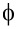) is approximately zero. For 
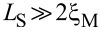
 the sawtooth profile at 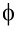 = π is preserved and robust when τ is reduced from the fully transparent to the tunnel regime, as seen in Figure 15b. Quite remarkably, in the tunneling regime, *I*(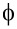) is finite away from *n*π for *n* = 0,1,…. The finite value of the supercurrent could serve as another indicator of the non-trivial topology and, thus, of the emergence of MBSs in the junction.

#### Disorder effects

Now we analyze the sawtooth profile of *I*(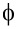) for *B > B*_c_ in the presence of disorder. Disorder is introduced as a random on-site potential *V_i_* in the tight-binding Hamiltonian given by Equation 4. The values of *V_i_* lie within [−*w*, *w*], with *w* being the disorder strength. When considering this kind of disorder, the chemical potential undergoes random fluctuations. Hence, values of *w* do not include 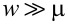.

**Figure 16:**
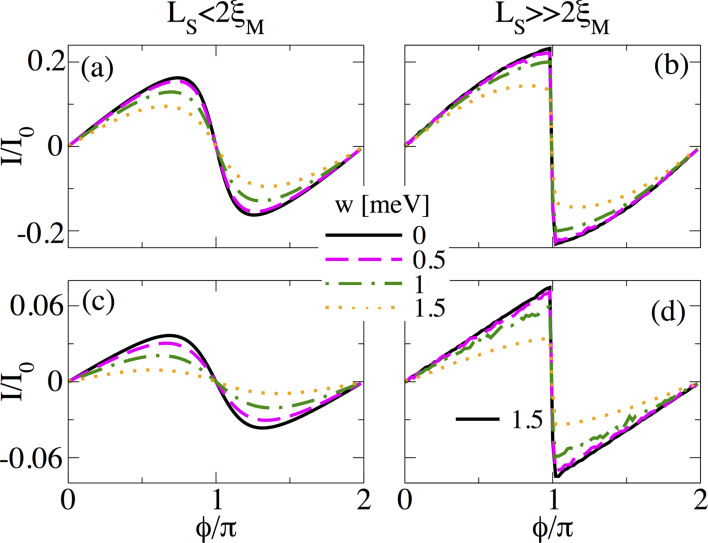
Effect of random on-site scalar disorder on the supercurrent *I*(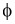) in (a,b) a short and (c,d) a long SNS junction at *B* = 1.5*B*_c_. (a,c) *L*_S_ = 2000 nm ≤ 2ξ_M_ and (b,d) *L*_S_ = 10000 nm 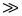 2ξ_M_. Each curve corresponds to 20 realizations of disorder, where *w* is the disorder strength. For small values of *w* of the order of the chemical potential, the sawtooth profile at 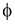 = π is preserved (see right panel). Parameters: *L*_N_ = 20 nm for short and *L*_N_ = 2000 nm for long junctions, α_R_ = 20 meV·nm, μ = 0.5 meV, Δ = 0.25 meV and 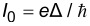.

In Figure 16(a,b) we present *I*(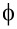) in short junctions at *B* = 1.5*B*_c_ for 20 disorder realizations and different values of the disorder strength *w*. Disorder of the order of the chemical potential μ has little effect on *I*(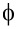) as shown by dashed curves in Figure 16a,b. The behavior of *I*(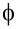) is approximately the same as without disorder. This reflects the robustness of the topological phase, and thus of MBSs, against fluctuations in the chemical potential [58–59]. Stronger disorder (dotted and dash-dot curves) reduce the maximum value of *I*(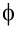) although its general behavior is preserved. The sawtooth profile at 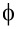 = π in Figure 16b is robust against moderate values of disorder strength. We have confirmed that these conclusions are still valid even when we consider disorder of the order of 5μ (not shown).

## Conclusion

In this numerical work we have performed a detailed investigation of the low-energy spectrum and supercurrents in short (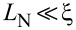) and long (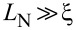) SNS junctions based on nanowires with Rashba SOC and in the presence of a Zeeman field.

In the first part, we have studied the evolution of the low-energy Andreev spectrum from the trivial phase into the topological phase and the emergence of MBSs in short and long SNS junctions. We have shown that the topological phase is characterized by the emergence of four MBSs in the junction (two at the outer part of the junction and two at the inner part) with important consequences to the equilibrium supercurrent. In fact, the outer MBSs are almost dispersionless with respect to superconducting phase 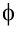, while the inner ones disperse and tend to reach zero at 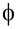 = π. A finite energy splitting at 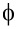 = π occurs when the length of the superconducting nanowire regions, *L*_S_, is comparable to or less than 2ξ_M_. Although in principle such energy splitting can be reduced by making the S regions longer, we conclude that in a system of finite length the current–phase curves are 2π-periodic and the splitting always spoils the so-called 4π-periodic fractional Josephson effect in an equilibrium situation.

In short junctions the four MBSs are truly bound within Δ only when 
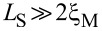
, while in long junctions the four MBSs coexist with additional levels, which profoundly affects phase-biased transport. As the Zeeman field increases in the trivial phase *B < B*_c_, the supercurrent *I*(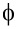) is reduced due to the reduction of the induced gap. In this case, the supercurrents *I*(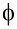) are independent of the length of the superconducting regions, *L*_S_, an effect preserved in both short and long junctions.

In short junctions in the topological phase with *B > B*_c_ the contribution of the four MBSs levels within the gap determines the shape of the current–phase curve *I*(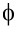) with only little contribution from the quasi-continuum. For *L*_S_ *<* 2ξ_M_, the overlap of MBS wavefunctions at each S region is finite, and the quasi-continuum contribution is appreciable and of the opposite sign than the contribution of the bound states. This induces a reduction of the maximum supercurrent in the topological phase. For 
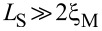
, when both the spatial overlap between MBSs and the splitting at 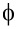 = π are negligible, the quasi-continuum contribution is very small and the supercurrent *I*(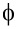) is dominated by the inner MBSs. Remarkably, we have demonstrated that the current–phase curve *I*(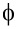) develops a clear sawtooth profile at 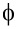 = π, which is independent of the quasi-continuum contribution and represents a robust signature of MBSs.

In the case of long junctions we have found that the additional levels that emerge within the gap affect the contribution of the individual MBSs. Here, it is the combined contribution of the levels within the gap and the quasi-continuum that determine the full current–phase curve *I*(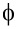), unlike in short junctions. The maximum supercurrent in long junctions is reduced in comparison to short junctions, as expected. Our results also show that the maximum value of the supercurrent in the topological phase depends on *L*_S_, acquiring larger values for 
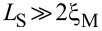
 than for *L*_S_ ≤ 2ξ_M_.

Finally, we have analyzed the robustness of the characteristic sawtooth profile in the topological phase against temperature, changes in transmission across the junction and random on-site scalar disorder. We found that a small finite temperature smooths it out due to thermal population of ABSs. We demonstrated that, although this might be a fragile indicator of MBSs, the fast sign change around 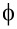 = π could help to distinguish the emergence of MBSs from trivial ABSs. Remarkably, the sawtooth profile is preserved against changes in transmission, i.e., it is preserved even in the tunneling regime. And finally, we showed that reasonable fluctuations in the chemical potential μ (up to 5μ) do not affect the sawtooth profile of *I*(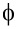) at 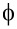 = π.

Our main contribution are summarized as follows. In short and long SNS junctions of finite length four MBSs emerge, two at the inner part of junction and two at the outer ends. The unavoidable overlap of the four MBSs gives rise to a finite energy splitting at 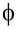 = π, thus rendering the equilibrium Josephson effect 2π-periodic in both short and long junctions. Current–phase curves of short and long junctions exhibit a clear sawtooth profile when the energy splitting near 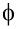 = π is small, which indicates the presence of weakly overlapping MBSs. Remarkably, the current–phase curves do not depend on *L*_S_ in the trivial phase for both short and long junctions, while they strongly depend on *L*_S_ in the topological phase. This effect is solely connected to the splitting of MBSs at 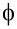 = π, indicating a unique feature of the topological phase and therefore of the presence of MBSs in the junction.

## Supporting Information

File 1 Majorana wavefunction and charge density in SNS junctions.
