# Supplementary material for: Andreev spectrum and supercurrents in nanowire-based SNS junctions containing Majorana bound states
Source: Beilstein J Nanotechnol. 2018 May 3;9:1339–57. doi: 10.3762/bjnano.9.127 (PMC6009489; doi:10.3762/bjnano.9.127)
Supplement: File 1 — Majorana wavefunction and charge density in SNS junctions. [file Beilstein_J_Nanotechnol-09-1339-s001.pdf]

Supporting Information

for

Andreev spectrum and supercurrents in nanowire-based SNS junctions containing Majorana bound states

Jorge Cayao[1], Annica M. Black-Schaffer[1], Elsa Prada[2] and Ramón Aguado*[3]

Address: [1]Department of Physics and Astronomy, Uppsala University, Box 516, S-751 20 Uppsala, Sweden; [2]Departamento de Física de la Materia Condensada, Condensed Matter Physics Center (IFIMAC) & Instituto Nicolás Cabrera, Universidad Autónoma de Madrid, E-28049 Madrid, Spain and [3]Instituto de Ciencia de Materiales de Madrid (ICMM-CSIC), Cantoblanco, 28049 Madrid, Spain

Email: Ramón Aguado - raguado@icmm.csic.es

* Corresponding author

Majorana wavefunction and charge density in SNS junctions

In the Supporting Information we provide calculations of the Majorana wavefunctions and charge density in order to support our findings in the main text of our manuscript.

Superconducting wire

From Equation 2 in the main manuscript text we can also calculate the wavefunctions associated to the energy levels after diagonalization. According to the chosen basis, they are obtained in the following form

$$\Psi(x) = \left(u_{\uparrow,i}, u_{\downarrow,i}, v_{\uparrow,i}, v_{\downarrow,i} \right)^T \tag{S1}$$

where T denotes the transpose operation, x denotes the site position i and N_S is the number of sites of the discretised superconducting nanowire. Then, the BdG wavefunction amplitude is given by

$$|\Psi(x)|^2 = |u_{\uparrow,i}|^2 + |u_{\downarrow,i}|^2 + |v_{\uparrow,i}|^2 + |v_{\downarrow,i}|^2 \,. \tag{S2}$$

Likewise, for the same price we can calculate the charge density as it was shown to provide useful information regarding MBSs [1]. It can be calculated by using the same information of $\Psi(x)$ and reads

$$|\rho(x)|^2 = |v_{\uparrow,i}|^2 + |v_{\downarrow,i}|^2 - |u_{\uparrow,i}|^2 - |u_{\downarrow,i}|^2 \,. \tag{S3}$$

Thus, the wavefunction amplitude and charge density can be calculated after finding $\Psi(x)$. Now, we calculate them associated to the two lowest energy levels of the topological superconducting nanowire. This is presented in Figure S1 for different lengths of the wire in the topological phase, where left and right columns correspond to the wavefunction amplitude and charge density, respectively.

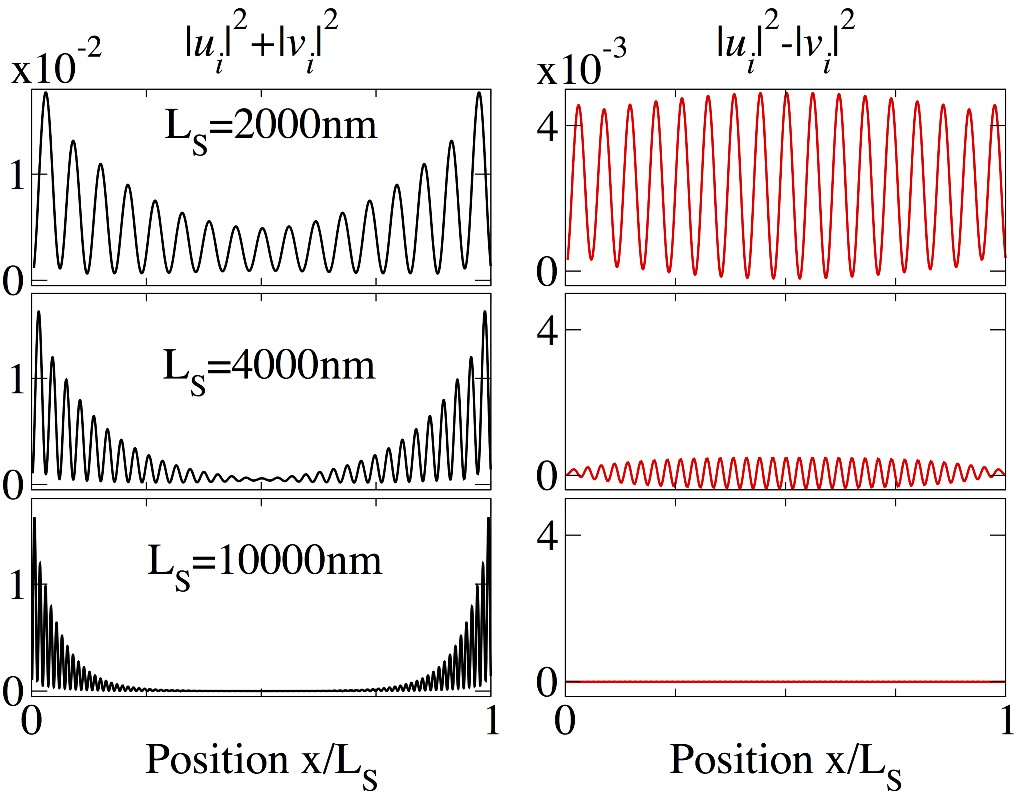

Figure S1: Wavefunction amplitude $|\Psi(x)|^2$ and charge density $|\rho(x)|^2$, given by Equation S2 and Equation S3, respectively, for different L_S corresponding to the two lowest levels (MBSs) in a topological superconducting nanowire. Parameters: $\alpha_R = 20\,\mathrm{meVnm}$, $\mu_N = \mu_S = 0.5\,\mathrm{meV}$, $\Delta = 0.25\,\mathrm{meV}$ and $B = 2B_c$.

Observe that for $L_S = 2000\,\mathrm{nm} < 2\xi_M$ (top left panel) $|\Psi(x)|^2$ of the two lowest levels decay from both ends into the bulk of the superconducting nanowire. Such levels exhibit an spatial overlap, which is reduced as L_S increases (see bottom left panels). On the other hand, when the spatial overlap of the Majorana wavefunction is finite, the charge density $|\rho(x)|^2$ develop an uniform oscillation pattern, predicted to be associated to MBSs [1]. As L_S increases, $|\rho(x)|^2$ gets reduced and reaches zero when $L_S \gg 2\xi_M$ (bottom right panel in Figure S1), signalling charge neutrality of the two lowest levels (MBSs).

SNS junction

In order to complete the analysis given in the main text, in this part we provide additional calculations for SNS junctions.

We present in Figure S2 and Figure S3 the BdG wave functions amplitude $|\Psi(x)|^2$ and charge density $|\rho(x)|^2$ of the MBSs in short and long junctions when the phase difference is $\phi = \pi$ so that the four MBSs are captured. These calculations are obtained following similar analysis as in the previous section for the Rashba nanowire.

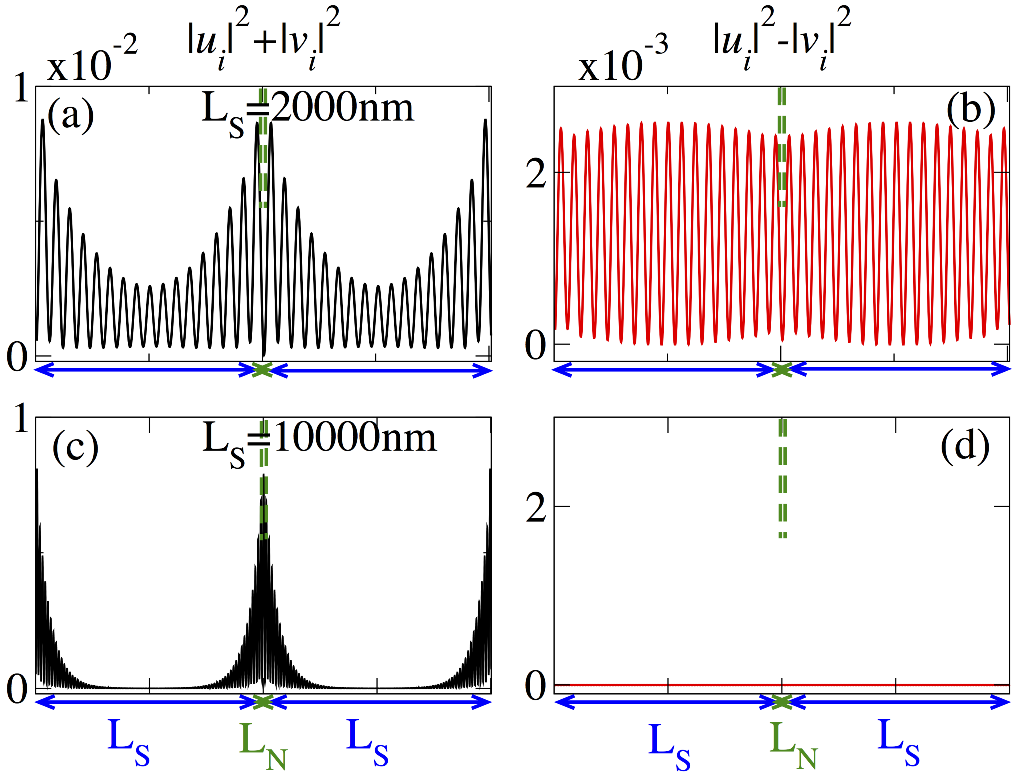

Figure S2: Wavefunction amplitude $|\Psi(x)|^2$ (a,c) and charge density $|\rho(x)|^2$ (b,d) in short junctions for $L_S \leq 2\xi_M$ (a,b) and $L_S \gg 2\xi_M$ (c,d) , corresponding to the two lowest levels (MBSs) in a topological superconducting nanowire. Parameters: $L_N = 20$ nm, $\alpha_R = 20$ meVnm, $\mu_N = \mu_S = 0.5$ meV, $\Delta = 0.25$ meV and $B = 2B_c$, $\phi = \pi$.

As observed in Figure S2a,c and Figure S3a,c, MBSs are localized at the ends of the S regions, exhibiting a considerable overlap when $L_S \leq 2\xi_M$ and a negligible one when $L_S \gg 2\xi_M$, as expected. In long junctions, Figure S3, an oscillating standing wave is developed in the normal region whose amplitude is smaller than the one in short junctions.

On the other hand, the associated charge density $|\rho(x)|$ exhibits uniform oscillations when the wave function overlap is finite, while it acquires zero value when the MBSs are located far apart, namely for $L_S \gg 2\xi_M$.

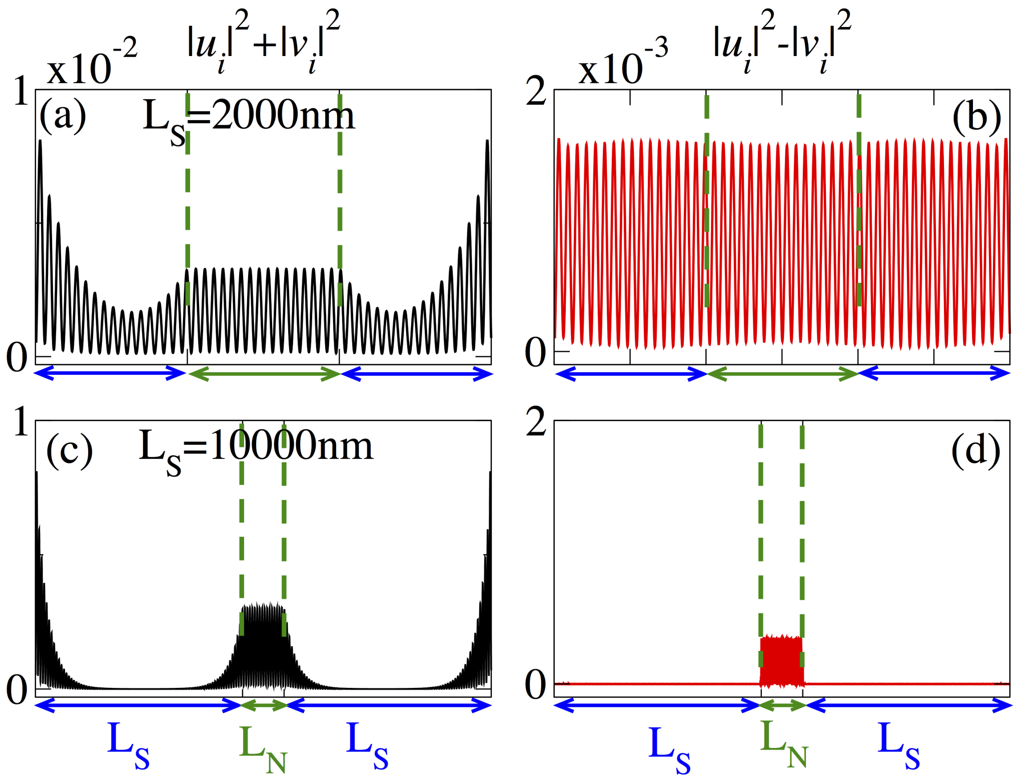

Figure S3: Same as in Figure S2 for a long junction with $L_{\mathrm{N}} = 2000\,\mathrm{nm}$.

References

1. Ben-Shach, G.; Haim, A.; Appelbaum, I.; Oreg, Y.; Yacoby, A.; Halperin, B. I. *Phys. Rev. B* **2015**, *91*, 045403. doi:10.1103/PhysRevB.91.045403.
